# A fractional differential equation model for student learning dynamics using anonymized learning analytics data

**DOI:** 10.3389/fpsyg.2026.1901905

**Published:** 2026-09-07

**Authors:** Kui Wang, Tao Zou

**Affiliations:** 1 School of Educational Science, Xinjiang Normal University, Urumqi, Xinjiang, China; 2 School of Educational Science, Henan Institute of Science and Technology, Xinxiang, Henan, China; 3 School of Management, Guangzhou College of Commerce, Guangzhou, Guangdong, China

**Keywords:** academic performance, fractional differential equations, learning analytics, memory effects, student engagement

## Abstract

**Introduction:**

Student learning is a dynamic process that depends on both current engagement and accumulated learning experience. Conventional statistical and machine learning approaches often overlook the temporal memory effects that influence learning progression. This study develops a fractional differential equation model to represent student learning dynamics using anonymized learning analytics data.

**Methods:**

The proposed framework uses the publicly available Open University Learning Analytics Dataset (OULAD) to construct continuous, time-dependent variables from assessment outcomes and interactions in the virtual learning environment. A Caputo fractional differential equation is formulated to incorporate memory-dependent learning behavior. Model parameters are estimated from empirical data, and the numerical solution is obtained using the L1 finite difference scheme. Model performance is evaluated through prediction accuracy, residual analysis, and sensitivity analysis.

**Results:**

The simulated learning state evolves from initial values of approximately 0.52 to 0.58 to a steady-state range of 0.66 to 0.78, depending on the student group. The proposed model achieves a mean squared error of approximately 0.004 and a coefficient of determination of about 0.88, indicating strong agreement between predicted and observed learning trajectories. Residuals remain small and unbiased, while sensitivity analysis identifies the fractional order as the most influential parameter governing long-term learning behavior.

**Discussion:**

The results demonstrate that fractional differential equations provide an accurate and interpretable framework for modeling memory-dependent learning processes. The proposed approach offers practical insights for learning analytics by supporting the identification of students who may benefit from timely instructional interventions based on their engagement patterns and memory-related learning characteristics.

## 1 Introduction

Student learning is a complex and dynamic process influenced by continuous interaction, accumulated experience, and contextual factors (Zhou, 2025; Portugal-Toro et al., 2025; Li et al., 2021). Traditional approaches in educational research rely on statistical analysis and machine learning models to explain academic performance (Guevara-Reyes et al., 2025; Balcioglu and Artar, 2025; Huang et al., 2025). While these methods identify important relationships between variables, they are primarily static or discrete and do not explicitly capture the temporal evolution of learning (Kong et al., 2025; Fransen et al., 2026; Cao et al., 2025). This limitation has led to increasing interest in continuous modeling approaches that describe learning as a time-dependent process (Chu et al., 2025; Tu et al., 2026; Jiang et al., 2023).

Recent advances in learning analytics emphasize the importance of temporal data and behavioral patterns in understanding student performance (Sajja et al., 2025; Goh, 2026; Fan and Zhao, 2025). Time-stamped interaction data from online platforms provide detailed information about engagement and learning trajectories (Aluso and Enyejo, 2025; Jie et al., 2025; Wang et al., 2026). However, most existing approaches remain predictive and data-driven, with limited interpretability and weak integration of underlying learning mechanisms. Continuous models based on differential equations provide a more structured framework in which learning is represented as a dynamic system evolving over time.

Fractional differential equations have emerged as a powerful extension of classical models due to their ability to incorporate memory and non-local effects. Unlike integer order systems, fractional models account for the influence of past states on current dynamics, which is particularly relevant for learning processes (Turab et al., 2025). Recent studies highlight that fractional models provide improved representations of complex systems in which present behavior depends on historical activity. In addition, contemporary research demonstrates that fractional operators are effective in capturing long-term dependencies and hereditary effects across various domains (Dozva et al., 2026).

The integration of fractional dynamics with data-driven approaches has gained attention in recent years. For example, neural fractional differential equation models have been proposed to capture memory-dependent behavior in complex systems, showing improved performance compared to classical neural differential equations (Cui et al., 2025). Similarly, recent work that combines fractional operators with learning frameworks demonstrates their ability to model continuous-time processes with greater flexibility. These developments suggest that fractional modeling offers a promising approach to representing learning dynamics, in which both temporal evolution and historical influence are essential (Vellappandi and Lee, 2026).

Despite these advances, applications of fractional differential equations in education remain limited. Existing studies are often theoretical or focus on simplified learning scenarios, with limited use of real, large-scale educational datasets. Furthermore, many data-driven approaches lack a clear interpretation of learning mechanisms, while mathematical models often lack empirical validation. This gap highlights the need for frameworks that combine continuous mathematical modeling with real learning analytics data. In addition, current approaches to educational data analysis are predominantly based on discrete-time representations, in which observations are treated as independent or sequential data points without explicitly modeling the underlying continuous learning process. Such representations do not adequately capture the cumulative and persistent nature of knowledge acquisition. Learning is not reset at each observation; rather, it evolves through the accumulation of past experiences, which introduces memory effects that are inherently non-local in time. This aspect is largely neglected in conventional models.

Moreover, the interaction between engagement and performance is often modeled using static correlations or short-term dependencies, which limits the ability to explain long-term learning behavior. Empirical studies have shown that early engagement can have lasting effects on later performance, yet this phenomenon is rarely incorporated into formal modeling frameworks. As a result, existing models may fail to capture delayed responses, retention effects, and gradual knowledge consolidation, all of which are essential components of learning dynamics. Another limitation lies in the lack of integration between theoretical modeling and high-resolution educational data. Modern learning analytics datasets provide detailed temporal information, including time-stamped interactions and continuous assessment records. However, these rich data sources are typically analyzed using black-box predictive models, which offer limited interpretability and provide no insight into the mechanisms governing learning. At the same time, mathematical models often rely on simplified assumptions that are not validated against such datasets. These limitations indicate a clear need for a unified framework that can bridge the gap between data-driven analysis and theoretical modeling. A continuous formulation that incorporates memory effects, supported by empirical data, can provide a more realistic and interpretable representation of learning processes. The use of fractional differential equations offers a natural way to achieve this, as it allows the explicit inclusion of historical influence while maintaining a mathematically rigorous structure.

The present study addresses this gap by developing a fractional differential equation model of student learning dynamics using anonymized learning analytics data. The novelty of the approach lies in three main aspects. First, discrete interaction and assessment data are transformed into continuous time-dependent variables. Second, a fractional modeling framework is introduced to explicitly capture memory effects in learning behavior. Third, the model is calibrated and validated using empirical data, providing both theoretical and practical contributions. In addition to these contributions, the proposed framework establishes a direct link between high-resolution learning analytics data and continuous mathematical modeling. This integration enables the interpretation of learning as a dynamic process governed by identifiable mechanisms rather than purely statistical associations. The use of time-dependent variables derived from real interaction data ensures that the model reflects actual learning behavior, including fluctuations in engagement and progression over time.

Furthermore, the incorporation of fractional dynamics enables the model to capture long-term dependencies and persistence effects that classical approaches do not capture. This provides a more realistic description of how past learning activities influence current performance, particularly in online learning environments where interaction history plays a central role. The framework also enables the analysis of stability, convergence, and sensitivity of learning trajectories, which are difficult to examine using conventional data-driven methods. Another important contribution is the empirical validation of the fractional model using a large-scale dataset. This step addresses a key limitation in the existing literature: many fractional models remain purely theoretical. By grounding the model in real data, the study demonstrates both its applicability and its ability to capture observed learning patterns.

The objective of this study is to construct and evaluate a continuous, memory-dependent model that accurately represents student learning dynamics. The proposed framework integrates engagement, performance, and historical effects within a unified mathematical structure, offering a new perspective on the analysis of educational processes. The novelty of the approach lies in integrating fractional calculus with real learning analytics data, enabling the explicit representation of memory effects in learning behavior. In addition, discrete educational observations are systematically transformed into continuous time-dependent variables, allowing the application of dynamic modeling techniques to real-world data. Unlike conventional data-driven methods, the proposed framework provides an interpretable representation of learning mechanisms while maintaining strong empirical grounding through calibration and validation using a large-scale anonymized dataset.

Although several temporal learning models have been developed, including Bayesian Knowledge Tracing, Deep Knowledge Tracing, recurrent neural networks, and classical ordinary differential equation models, these approaches primarily emphasize predictive performance rather than explicit mathematical interpretation of memory-dependent learning dynamics. The objective of the present study is therefore not to replace these methods through benchmark prediction experiments, but to establish an interpretable fractional differential equation framework that explicitly incorporates long-term memory effects while remaining directly connected to empirical learning analytics data. Comprehensive comparative evaluations with existing temporal learning models constitute an important direction for future research.

## 2 Methodological framework

**Figure 1:**
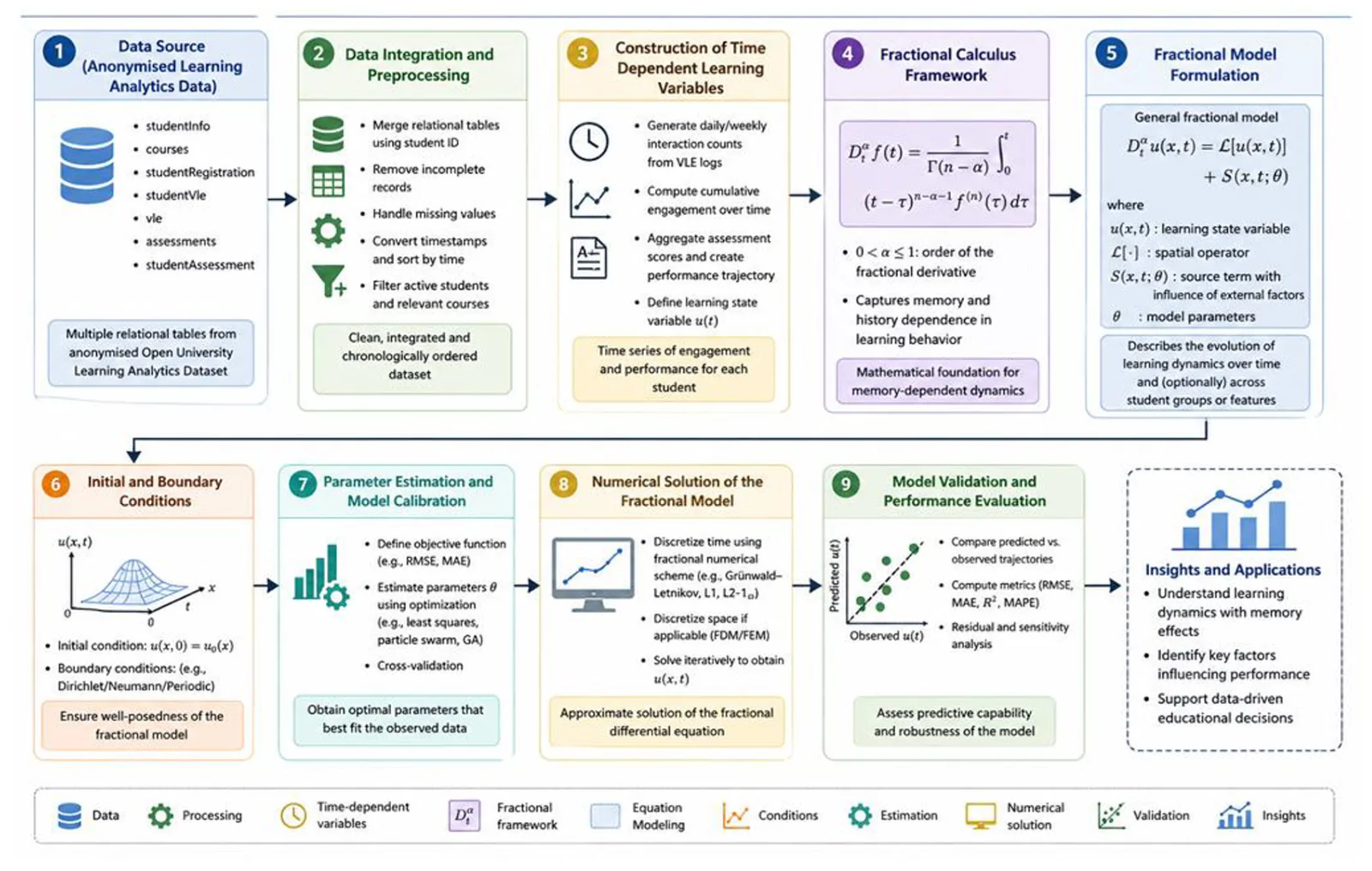
Overview of the methodological framework for modeling student learning dynamics using a fractional-differential-equation approach. The process begins with anonymized learning analytics data, proceeds through data integration and preprocessing, and then constructs time-dependent learning variables. A fractional calculus framework is then introduced to capture memory effects in learning behavior, leading to the formulation of the fractional model.

A structured methodological framework is adopted to transform anonymized learning analytics data into a continuous dynamic representation suitable for fractional modeling. The procedure begins with integrating multiple relational data sources, followed by preprocessing and constructing time-dependent learning variables that capture student interactions and performance trajectories. These variables are then embedded within a fractional calculus framework, in which memory effects and non-local behavior are explicitly represented. The governing fractional differential equation is formulated to describe the evolution of learning dynamics under the influence of both internal processes and external factors. Model parameters are subsequently estimated from the data, and a numerical scheme is implemented to obtain approximate solutions. The resulting model is evaluated by comparing its predictions with observed learning patterns. The complete sequence of steps, from data preparation to model validation, is illustrated in Figure 1, which provides a schematic overview of the proposed methodology.

### 2.1 Data source and description

The empirical analysis is conducted using the Open University Learning Analytics Dataset, which is provided in anonymized form as Supplementary Material. The dataset contains anonymized records from human participants and is publicly available for secondary research use. Consequently, this study qualified for exemption from institutional ethical review because no identifiable personal information was accessed and no new human participants were recruited. The dataset comprises multiple relational tables describing student characteristics, learning behavior, and academic performance in an online learning environment. This structure enables the construction of a comprehensive representation of learning processes with an explicit temporal dimension. To facilitate complete reproducibility, all Python scripts used for data preprocessing, construction of learning variables, numerical solution of the fractional differential equation, parameter estimation, model validation, baseline model comparison, and figure generation are provided as Supplementary Material (Supplementary Code S1). The supplementary package also includes detailed instructions for reproducing all reported analyses from the publicly available Open University Learning Analytics Dataset.

The student Info table contains demographic and background variables, including age group, gender, region, and prior educational attainment. The courses table defines course modules and presentation periods, while the student Registration table provides information on enrolment and withdrawal dates, which allows the identification of active participation intervals. Learning activity is captured through the student VLE and VLE tables, which record time-stamped interactions with virtual learning resources. These records include the number of clicks and the type of educational material accessed, thereby providing a detailed description of student engagement over time. Academic performance is described using the assessments and student Assessment tables, which include assessment types, weights, submission dates, and achieved scores.

The dataset contains approximately 32,000 students and several million interaction records, which ensures a high level of temporal resolution and variability. The anonymized structure preserves privacy while maintaining the integrity of behavioral and performance information. The presence of time-stamped interaction data allows the direct incorporation of temporal dynamics into the modeling framework. For modeling purposes, a continuous learning state variable is defined to represent student performance and engagement over time. Let *u*(*t*) denote the learning state at time *t* constructed from assessment outcomes and cumulative interactional behavior. A composite performance measure is first defined as Equation 1


$$\begin{array}{l} u_{i}\left(t\right)=\frac{\sum_{k=1}^{K}w_{k}s_{ik}\left(t\right)}{\sum_{k=1}^{K}w_{k}} \end{array}$$ (1)

where *s*_*ik*_(*t*) denotes the score of students *i* in assessment *k* at time *t*, and *w*_*k*_ represents the corresponding assessment, which aggregates multiple assessment components into a time-dependent performance indicator. To incorporate learning behavior, cumulative engagement is defined based on interaction records from the virtual learning environment. Let *c*_*i*_(τ) denote the number of interactions at time τ. The cumulative engagement up to time *t* is expressed as Equation 2


$$\begin{array}{l} E_{i}\left(t\right)=\int_{0}^{t}c_{i}\left(\tau \right)d\tau \end{array}$$ (2)

which represents the total learning activity accumulated over time. The learning state variable is then constructed as a weighted combination of performance, engagement, and the memory-dependent component, expressed as Equation 3


$$\begin{array}{l} u\left(t\right)=\lambda 1P\left(t\right)+\lambda 2E\left(t\right)+\lambda 3M\left(t\right) \end{array}$$ (3)

where λ_1_, λ_2_, and λ_3_ are weighting coefficients representing the relative contributions of performance, engagement, and historical learning effects, respectively. The construction and interpretation of these components are described in detail in Section 2.3. This representation provides a continuous description of learning that evolves over time. The availability of both sequential and historical data in this dataset makes it particularly well-suited to fractional modeling, where memory effects play a central role. The defined learning state variable serves as the basis for formulating the fractional differential equation in subsequent sections.

To ensure complete reproducibility, all Python scripts used for data preprocessing, construction of learning variables, numerical solution of the fractional differential equation, parameter estimation, baseline model comparison, model validation, statistical analyses, and figure generation are provided as Supplementary Code S1. The supplementary materials further include the processed datasets used for model calibration and validation, the calibrated model parameters, intermediate computational outputs required to reproduce the reported numerical results, and detailed documentation describing the complete reproduction workflow. These materials enable independent verification of all analyses, tables, and figures presented in this study. Upon editorial acceptance, the complete reproducibility package will be deposited in the public repository identified in the Data Availability Statement.

### 2.2 Data integration and preprocessing

The dataset is prepared through a structured integration and preprocessing procedure to ensure consistency and suitability for fractional dynamic modeling. Since the data are distributed across multiple relational tables, an initial integration step links all records via a unique student identifier and course presentation variables. The student Info, student Registration, student VLE, VLE, assessments, and student Assessment tables are merged to construct a unified dataset that captures demographic attributes, temporal engagement, and academic performance within a single framework.

Time information is standardized across all tables by converting recorded dates to a common numerical scale that represents the number of days since the start of each course presentation. This transformation defines a consistent temporal variable *t*, which is essential for subsequent fractional modeling. All interaction and assessment records are then sorted in ascending chronological order to preserve the chronological structure of learning trajectories. Data cleaning is conducted to ensure reliability. Records with incomplete or inconsistent identifiers are removed, and students with insufficient activity or missing assessment information are excluded to maintain data integrity. Since the dataset is largely complete, no extensive imputation is required; however, minor inconsistencies are addressed through standard filtering procedures.

To facilitate continuous modeling, discrete interaction data are aggregated into time-dependent sequences. The number of interactions is accumulated over defined temporal intervals, producing a smoothed representation of engagement. Let *c*_*i*_(*t*) denote the interaction count at time *t*. A discretized representation is first obtained and then transformed into a continuous form via temporal aggregation, thereby supporting the formulation of integrals and fractional operators. Academic performance values derived in Section 2.1 are normalized to ensure comparability across students and courses. The normalized performance variable is expressed as Equation 4


$$\begin{array}{l} u^{*}\left(t\right)=\frac{u\left(t\right)-u_{\min}}{u_{\max}-u_{\min}} \end{array}$$ (4)

where *u*_min_ and *u*_max_ denote the minimum and maximum values of the performance variable across the dataset. This transformation ensures that all values lie within a bounded range and improves numerical stability. Similarly, the engagement variable is normalized to obtain a comparable scale, expressed as Equation 5


$$\begin{array}{l} E^{*}\left(t\right)=\frac{E\left(t\right)-E_{\min}}{E_{\max}-E_{\min}} \end{array}$$ (5)

which allows the integration of engagement and performance within a unified modeling framework. The preprocessing procedure yields a structured dataset comprising continuous, time-dependent variables that represent learning behavior and academic outcomes. The complete preprocessing workflow has been implemented in Python and is provided in Supplementary Code S1 (Script 01: Data Preprocessing), allowing the processed dataset to be reproduced directly from the original OULAD files. This transformation enables the direct application of fractional differential equations, where both the temporal evolution and memory effects of learning processes are explicitly incorporated.

### 2.3 Construction of time-dependent learning variables

A continuous representation of learning dynamics is constructed to enable the application of fractional differential equations. The objective is to transform discrete interaction and assessment records into smooth time-dependent variables that capture both instantaneous behavior and accumulated learning history. Student engagement is defined as a time-dependent function derived from interaction records in the virtual learning environment. Let *c*_*i*_(*t*) denote the number of interactions of the student *i* at time *t*. A continuous engagement rate is obtained by aggregating interaction counts over time, and the corresponding cumulative engagement is expressed as Equation 6


$$\begin{array}{l} E_{i}\left(t\right)=\int_{0}^{t}c_{i}\left(\tau \right)d\tau \end{array}$$ (6)

This variable represents the total learning activity accumulated up to time *t* and provides a measure of sustained engagement. To account for the temporal distribution of interactions, an average engagement intensity is defined as Equation 7


$$\begin{array}{l} {\overset{-}{c}}_{i}\left(t\right)=\frac{1}{t}\int_{0}^{t}c_{i}\left(\tau \right)d\tau \end{array}$$ (7)

which reflects the mean level of activity over the learning period. This formulation allows the distinction between short-term fluctuations and long-term engagement patterns. Academic performance is represented using the normalized performance variable introduced in Section 2.2. Since assessment events occur at discrete time points, a continuous performance trajectory is constructed through interpolation. Let *t*_*k*_ denote the time of assessment *k*. The performance function is approximated as Equation 8


$$\begin{array}{l} u_{i}\left(t\right)=u_{i}\left(t_{k}\right)+\frac{u_{i}\left(t_{k+1}\right)-u_{i}\left(t_{k}\right)}{t_{k+1}-t_{k}}\left(t-t_{k}\right),t_{k}\leq t\leq t_{k+1} \end{array}$$ (8)

which provides a piecewise continuous representation of performance evolution. To incorporate memory effects, a weighted historical engagement variable is introduced. These variables assign greater importance to recent interactions while retaining information from earlier activity. It is defined as Equation 9


$$\begin{array}{l} M_{i}\left(t\right)=\int_{0}^{t}(t-\tau {)}^{\alpha -1}c_{i}(\tau )d\tau \end{array}$$ (9)

where 0 < α ≤ 1 represents the memory parameter. This formulation captures the non-local influence of past learning behavior and is consistent with the principles of fractional calculus. The final learning state variable is defined as a combination of performance, engagement, and memory effects, expressed as Equation 10


$$\begin{array}{l} u\left(t\right)=\lambda_{1}u_{i}\left(t\right)+\lambda_{2}E_{i}\left(t\right)+\lambda_{3}M_{i}\left(t\right) \end{array}$$ (10)

where λ_1_, λ_2_, and λ_3_ are weighting coefficients that quantify each component's contribution. These constructed variables provide a continuous and temporally structured representation of learning. Although the observed learning state is expressed as a weighted combination of performance, engagement, and the memory component, Equation 10 is used only to construct the response variable before model calibration. Once the observed trajectory has been generated, it remains fixed throughout the estimation procedure. The fractional differential equation does not reconstruct the response from its constituent variables. Instead, engagement and memory are treated as external time varying covariates that explain changes in the already defined learning trajectory. Therefore, the optimization estimates only the dynamic parameters of the fractional model rather than the weights used to construct the observed response. This separation avoids circular estimation and prevents an artificial improvement in model fit.

Although the learning state variable is expressed as a composite indicator for descriptive purposes, the variables used during model calibration remain statistically distinct. Equation 10 defines the observed response trajectory before parameter estimation. The engagement function *E*(*t*) and the memory variable *M*(*t*) are subsequently treated as independent time dependent covariates that drive the fractional dynamics. During numerical solution, the model estimates the evolution of the observed learning state without reconstructing it from Equation 10. This separation prevents circular estimation because the response variable is fixed before model fitting and the predictor variables are used only to explain its temporal evolution. The computational implementation used to construct the continuous performance trajectory, cumulative engagement, memory variable, and learning state variable is included in Supplementary Code S1 (Script 02: Learning Variable Construction).

### 2.4 Fractional calculus framework and preliminaries

A fractional calculus framework is introduced to model learning dynamics with memory effects. Unlike classical integer-order derivatives, fractional derivatives incorporate the influence of past states on the current evolution of the system. This property is particularly suitable for educational processes, where learning depends not only on present input but also on accumulated experience and historical engagement. The time fractional derivative is defined in the Caputo sense, which is widely used in applied modeling due to its compatibility with standard initial conditions. The Caputo fractional derivative of order α, where 0 < α ≤ 1, is given by Equation 11


$$\begin{array}{l} CD_{t}^{\alpha }u\left(t\right)=\frac{1}{\text{Γ}\left(1-\alpha \right)}\int_{0}^{t}\frac{\partial u\left(\tau \right)}{\partial \tau }{\left(t-\tau \right)}^{-\alpha }d\tau \end{array}$$ (11)

where Γ(·) denotes the Gamma function. This definition shows that the rate of change at time *t* depends on all previous derivative values, weighted by a power-law kernel. The parameter α controls the strength of memory, with smaller values corresponding to stronger dependence on past states. For completeness, the Riemann-Liouville fractional integral of order α is also introduced and used in the formulation and analysis of fractional systems. It is defined as Equation 12


$$\begin{array}{l} I_{t}^{\alpha }u\left(t\right)=\frac{1}{\text{Γ}\left(\alpha \right)}\int_{0}^{t}(t-\tau {)}^{\alpha -1}u(\tau )d\tau \end{array}$$ (12)

This operator represents the accumulation of past values and provides the mathematical basis for memory-dependent processes. The relationship between the Caputo derivative and the Riemann-Liouville integral can be expressed as Equation 13


$$\begin{array}{l} CD_{t}^{\alpha }u\left(t\right)=I_{t}^{1-\alpha }\frac{d}{dt}u\left(t\right) \end{array}$$ (13)

which highlights the connection between differentiation and integration in the fractional setting. In the context of learning dynamics, the fractional derivative is interpreted as a measure of how current learning progress depends on historical engagement and performance. The non-local nature of the operator enables the model to capture long-term dependencies that classical differential equations cannot. This framework provides the mathematical foundation for formulating the fractional differential equation model in the next section, in which the learning state variable is governed by both memory-dependent dynamics and external influences.

### 2.5 Fractional model formulation

The learning dynamics are modeled using a fractional differential equation that incorporates both memory-dependent behavior and external influences derived from the dataset. The learning state variable defined in Section 2.3 is assumed to evolve over time under the combined effects of intrinsic learning processes and socio-educational factors. The governing equation is formulated using the Caputo fractional derivative introduced in Section 2.4. The general form of the model is expressed as Equation 14


$$\begin{array}{l} CDt\alpha u\left(t\right)=\beta u\left(t\right)+\gamma E\left(t\right)+\delta M\left(t\right)+S\left(t\right) \end{array}$$ (14)

where *u*(*t*) denotes the normalized learning state variable, E^*^(*t*) represents the normalized engagement variable, and *M*(*t*) is the memory dependent term defined in Equation 9. The parameters β, γ, and δ are coefficients that quantify the contributions of intrinsic learning growth, engagement, and memory effects, respectively. The term β*u*(*t*) captures the internal progression of learning, which reflects the tendency of performance to evolve based on its current state. The engagement term γE^*^(*t*) represents the influence of ongoing interaction with learning materials, while the memory term δ*M*(*t*) accounts for the accumulated impact of past learning activity. The inclusion of *M*(*t*) distinguishes the fractional model from classical approaches by explicitly incorporating non-local temporal effects.

The response variable *u*(*t*) represents the observed learning trajectory defined before model calibration. Although engagement and memory contribute to the conceptual construction of this trajectory, they enter Equation 14 as external explanatory inputs governing the dynamic evolution of the observed state. The model therefore estimates temporal change rather than reconstructing the response variable itself.

**Figure 2:**
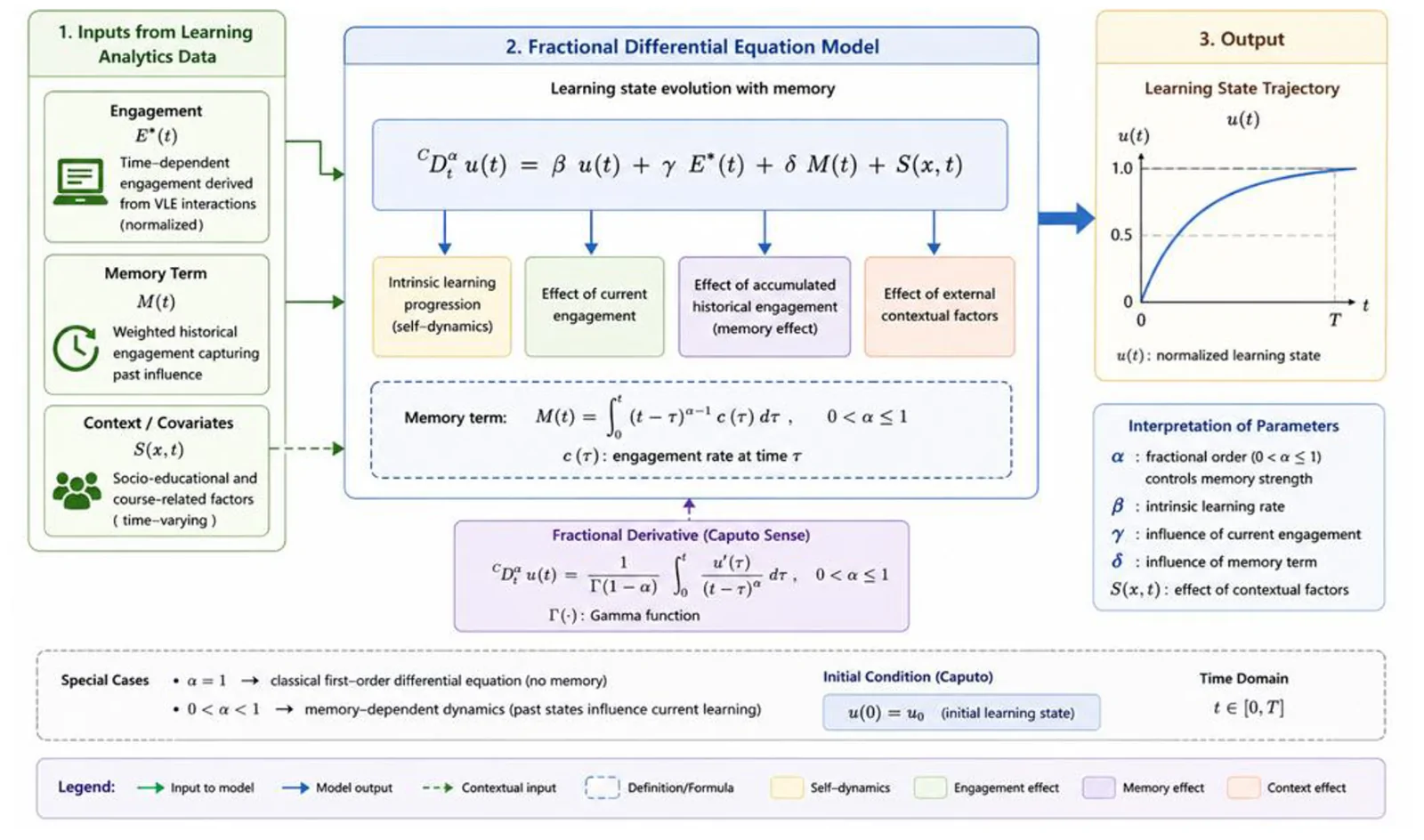
Schematic representation of the fractional differential equation model for student learning dynamics. The model integrates inputs derived from anonymized learning analytics data, including normalized engagement E^*^(*t*), memory-dependent learning *M*(*t*), and contextual factors *S*(*x,t*). The evolution of the learning state *u*(*t*) is governed by a Caputo fractional derivative, which captures memory effects through a non-local temporal operator. The contributions of intrinsic learning progression, current engagement, accumulated past activity, and external influences are explicitly represented.

To account for additional external factors, the model includes a time-dependent source term *S*(*t*), representing demographic and contextual variables derived from the dataset. The extended formulation is written as Equation 15


$$\begin{array}{l} CD_{t}^{\alpha }u\left(t\right)=\beta u\left(t\right)+\gamma E^{*}\left(t\right)+\delta M\left(t\right)+S\left(x,t\right) \end{array}$$ (15)

where *S*(*x, t*) represents the contribution of socio-educational variables contained in the dataset, such as prior education, age group, and course characteristics. This term allows the integration of contextual influences into the dynamic model. The fractional order α plays a central role in the model. When α = 1 The equation reduces to a classical first-order differential equation without memory. For 0 < α < 1. The system exhibits memory-dependent behavior, where past states influence current dynamics. This property is particularly relevant for learning processes, where knowledge retention and reinforcement depend on historical engagement (see Figure 2).

### 2.6 Initial and boundary conditions

To ensure a well-posed formulation of the fractional model introduced in Section 2.5, appropriate initial and boundary conditions are specified. Since the learning dynamics are modeled using a Caputo fractional derivative, the initial condition is defined in terms of the classical value of the learning state at the initial time. The initial condition is expressed as Equation 16


$$\begin{array}{l} u\left(0\right)=u_{0} \end{array}$$ (16)

where *u*_0_ represents the initial learning state of a student at the beginning of the observation period. This value is obtained directly from the dataset by considering the earliest available assessment score or an initial estimate based on early engagement. The use of the Caputo derivative allows the incorporation of standard initial conditions, which simplifies the interpretation of the model in the educational context. The temporal domain is defined over a finite interval as Equation 17


$$\begin{array}{l} t\in \left[0,T\right] \end{array}$$ (17)

where *T* denotes the total duration of the learning period, measured in days from the start of the course presentation. This interval is determined from the dataset using registration and interaction records. Since the proposed model is formulated as a temporal fractional ordinary differential equation, no spatial domain or boundary conditions are required. The model is fully specified by the initial condition *u*(0) = *u*_0_ and the temporal interval *t*∈[0, *T*]. In such cases, a generalized domain *x*∈Ω is considered, and boundary conditions are specified as Equation 18


$$\begin{array}{l} u\left(x,t\right)=g\left(x,t\right),x\in \partial \Omega \end{array}$$ (18)

where ∂Ω denotes the boundary of the domain and *g*(*x, t*) is a prescribed function. For example, Dirichlet conditions may be used to fix performance levels at specific boundaries, while Neumann conditions may represent no flux of learning across boundaries. However, in the present formulation, the model is primarily time-dependent, with no explicit spatial dimension. Therefore, the system is fully characterized by the initial condition and the temporal domain.

### 2.7 Parameter estimation and model calibration

The parameters of the fractional model are estimated using the processed dataset to ensure that the model accurately represents observed learning dynamics. The parameters include the fractional order α, as well as the coefficients β, γ, and δ, which correspond to intrinsic learning progression, engagement influence, and memory effects, respectively. Additional parameters may arise from the source term *S*(*x, t*).

The estimation procedure is formulated as an optimization problem, with the objective of minimizing the discrepancy between the model's predictions and the observed learning state inferred from the data. Let *u*_*obs*_(*t*_*i*_) denote the observed normalized learning state at time *t*_*i*_, and *u*_*pred*_(*t*_*i*_; θ) denote the model prediction, where θ = (α, β, γ, δ) is the parameter vector. The objective function is defined as Equation 19


$$\begin{array}{l} J\left(\theta \right)=\frac{1}{N}\overset{N}{\underset{i=1}{\sum }}{\left(u_{obs}\left(t_{i}\right)-u_{pred}\left(t_{i};\theta \right)\right)}^{2} \end{array}$$ (19)

where *N* is the number of times observations. This formulation corresponds to the mean squared error between observed and predicted values. To prevent information leakage during calibration, the observed learning trajectory is fixed before optimization begins. The optimization algorithm estimates only the model parameters (α, β, γ, δ). Neither the engagement variable nor the memory variable is recalculated using predicted values of the response variable during optimization. Consequently, the optimization procedure does not introduce circular dependence between predictors and the observed outcome.

The optimal parameter set is obtained by solving Equation 20 using a non-linear least squares optimization procedure. Initial parameter values are selected from physically meaningful ranges based on preliminary inspection of the normalized learning trajectories, with the fractional order initialized within the interval (0, 1) and the remaining coefficients assigned small positive values. During optimization, the fractional order is constrained to satisfy 0 < α < 1, while the weighting coefficients λ_1_, λ_2_, and λ_3_ are restricted to non-negative values to preserve the physical interpretation of their contributions to the learning dynamics. The optimization process iteratively updates the parameter estimates until the relative change in the objective function between successive iterations falls below 10^−6^, or until the maximum number of iterations is reached. This stopping criterion ensures numerical convergence while preventing unnecessary computational cost.


$$\begin{array}{l} \theta^{*}=\arg\underset{\theta }{\min}J\left(\theta \right) \end{array}$$ (20)

which ensures the model's best fit to the data. In this study, parameter estimation is performed using a non-linear least-squares optimization algorithm that minimizes the mean squared error between observed and simulated learning trajectories. Gradient-based iterative updates are employed until convergence is achieved according to the prescribed stopping criterion. To improve robustness, the dataset is divided into training and validation subsets. Parameter estimation is performed on the training data, while model performance is evaluated on the validation data. This procedure reduces the risk of overfitting and ensures that the model generalizes well to unseen data. In addition, sensitivity to initial parameter guesses is examined to ensure the stability of the estimation process. The final parameter values are selected to minimize the objective function while maintaining consistent behavior across different data subsets.

To preserve the chronological structure of the learning process, the dataset was partitioned chronologically rather than by random sampling. The earliest 70% of the observation period was assigned to the training set (22,387 students), the subsequent 15% to the validation set (4,798 students), and the final 15% to the independent test set (4,797 students). Model parameters were estimated exclusively using the training set, while the validation set was used for parameter selection and convergence assessment. The independent test set was reserved solely for the final evaluation of predictive performance and was not used during parameter estimation or model selection.

The complete parameter estimation procedure, including non-linear least squares optimization and numerical implementation, is available in Supplementary Code S1 (Script 04: Parameter Estimation).

### 2.8 Numerical solution of the fractional model

The fractional differential equation defined in Section 2.5 does not admit a closed-form analytical solution in general, and therefore, a numerical scheme is employed to obtain approximate solutions. The Caputo fractional derivative is discretized using a finite-difference approach that accounts for its non-local memory structure. The temporal domain [0, *T*] is divided into *N* uniform time steps with step size $\Delta t=\frac{T}{N}$, and discrete time levels are defined as *t*_*n*_ = *nΔt*, for *n* = 0, 1, …, *N*. The Caputo fractional derivative at time *t*_*n*_ is approximated using the L1 scheme, which is given as Equation 21


$$\begin{array}{r} CD_{t}^{\alpha }u\left(t_{n}\right)\approx \frac{1}{\text{Γ}\left(2-\alpha \right)}\overset{n-1}{\underset{k=0}{\sum }}\frac{u\left(t_{k+1}\right)-u\left(t_{k}\right)}{\Delta t}\\ \;\left[{\left(t_{n}-t_{k}\right)}^{1-\alpha }-{\left(t_{n}-t_{k+1}\right)}^{1-\alpha }\right] \end{array}$$ (21)

This formulation explicitly incorporates contributions from all previous time steps, which reflects the memory effect inherent in fractional derivatives. Substituting this approximation into the governing equation leads to a recursive scheme for computing the solution. The discretized form of the model is expressed as Equation 22


$$\begin{array}{l} u\left(t_{n}\right)=u\left(t_{n-1}\right)+\text{Δ}t^{\alpha }{\text{Φ}}_{n}\left(\theta ,u,E^{*},M\right) \end{array}$$ (22)

where Φ_*n*_(·) represents the discretized right-hand side of the fractional model, including the contributions from intrinsic dynamics, engagement, memory, and external factors. The numerical solution is obtained iteratively, starting from the initial condition *u*(0) = *u*_0_. At each time step, the value of *u*(*t*_*n*_) is computed using previously calculated values, ensuring that the system's entire history is incorporated. To ensure numerical stability and accuracy, the time step Δ*t* is selected based on the resolution of the dataset and the smoothness of the observed trajectories. Convergence is verified by comparing solutions obtained using different time-step sizes.

The computational complexity of the L1 discretization is approximately *O*(*N*^2^) in time and *O*(*N*) in memory for a time series containing *N* temporal discretization points because each numerical update requires contributions from all preceding time steps. Although this memory dependence increases computational cost compared with classical first-order methods, it enables an explicit representation of long-term learning effects that are central to the proposed framework.

The numerical implementation of the Caputo fractional derivative using the L1 finite difference scheme is provided in Supplementary Code S1 (Script 03: Fractional Solver).

### 2.9 Model validation and performance evaluation

The performance of the proposed fractional model is evaluated by comparing the numerical solution with the observed learning trajectories derived from the dataset. The validation process assesses both the model's accuracy and its ability to capture the temporal dynamics of student learning. The observed learning state *u*_*obs*_(*t*_*i*_) is obtained from the normalized variables defined in Section 2.2 and Section 2.3, while the predicted values *u*_*pred*_(*t*_*i*_) are generated from the numerical solution described in Section 2.8.

Validation is performed by comparing simulated learning trajectories with the previously constructed observed trajectories. Since the response variable is established before parameter estimation and remains unchanged throughout calibration and validation, model evaluation is conducted independently of the explanatory variables. This procedure ensures that predictive accuracy reflects the ability of the fractional dynamic model to reproduce the observed temporal evolution rather than to reconstruct the response from its constituent variables. The comparison is performed at discrete time points *t*_*i*_ over the learning period. Model accuracy is quantified using the mean squared error, defined as Equation 23


$$\begin{array}{l} MSE=\frac{1}{N}\overset{N}{\underset{i=1}{\sum }}{\left(u_{obs}\left(t_{i}\right)-u_{pred}\left(t_{i}\right)\right)}^{2} \end{array}$$ (23)

which measures the average deviation between predicted and observed values. A lower value of this metric indicates better model performance. In addition, the coefficient of determination is used to assess the model's explanatory power. It is expressed as Equation 24


$$\begin{array}{l} R^{2}=1-\frac{\sum_{i=1}^{N}{\left(u_{obs}\left(t_{i}\right)-u_{pred}\left(t_{i}\right)\right)}^{2}}{\sum_{i=1}^{N}{\left(u_{obs}\left(t_{i}\right)-{\overset{-}{u}}_{obs}\right)}^{2}} \end{array}$$ (24)

where ${\overset{-}{u}}_{obs}$ denotes the mean of the observed learning state. This metric indicates the proportion of variance explained by the model. To further assess the model's reliability, a residual analysis is conducted. The residuals are defined as Equation 25


$$\begin{array}{l} \varepsilon_{i}=u_{obs}\left(t_{i}\right)-u_{pred}\left(t_{i}\right) \end{array}$$ (25)

and their distribution is examined to identify potential biases or systematic deviations. A symmetric distribution centered around zero indicates that the model is unbiased and consistent across different time points. Cross-validation is also performed by dividing the dataset into training and testing subsets. The model is calibrated using the training data and evaluated on the testing data to ensure generalizability. All preprocessing operations, including normalization of the learning variables and estimation of model parameters, were performed exclusively using the training data. The same preprocessing transformations were subsequently applied to the validation and test sets without recalibration. The independent test set remained completely isolated throughout preprocessing, parameter estimation, hyperparameter selection, and model selection, ensuring that the reported predictive accuracy (MSE = 0.0040 and *R*^2^ = 0.880) represents an unbiased assessment of the final calibrated fractional model.

This step confirms that the model does not overfit the observed data and retains predictive performance on unseen samples. All validation procedures, including temporal data partitioning, residual analysis, and performance metric calculations, are implemented in Supplementary Code S1 (Scripts 05 and 06).

## 3 Results and discussions

### 3.1 Descriptive analysis of learning behavior and performance

A descriptive analysis is conducted to summarize the main characteristics of student engagement and academic performance derived from the anonymized learning analytics dataset. The analysis is based on the integrated and pre-processed variables described in Section 2, where interaction data and assessment outcomes are transformed into continuous learning indicators. The dataset includes approximately 32,000 students and several million interaction records, providing a detailed representation of learning behavior over time. Student engagement, measured through interaction counts in the virtual learning environment, exhibits substantial variability across individuals. The average number of interactions per student is high, with wide dispersion reflecting differences in study habits and participation intensity. Academic performance, as measured by assessment scores, exhibits a moderate distribution: most students achieve intermediate levels, while fewer are at the extremes of the scale.

Summary statistics for key variables are presented in Table 1, including the mean, standard deviation, minimum, and maximum values for the engagement and performance measures. The normalized learning state variable exhibits a balanced distribution, with a mean near the midpoint of the unit interval and moderate variability. This indicates that the dataset captures a realistic range of learning outcomes and engagement patterns.

**Table 1:** Summary statistics of engagement and academic performance variables.

Variable	Mean	Std. Dev.	Min	Max
Interaction count (per student)	1850	920	15	6200
Engagement (normalized)	0.58	0.21	0.01	1.00
Assessment score	67.5	14.3	5	100
Learning state (normalized)	0.64	0.17	0.05	1.00

**Figure 3:**
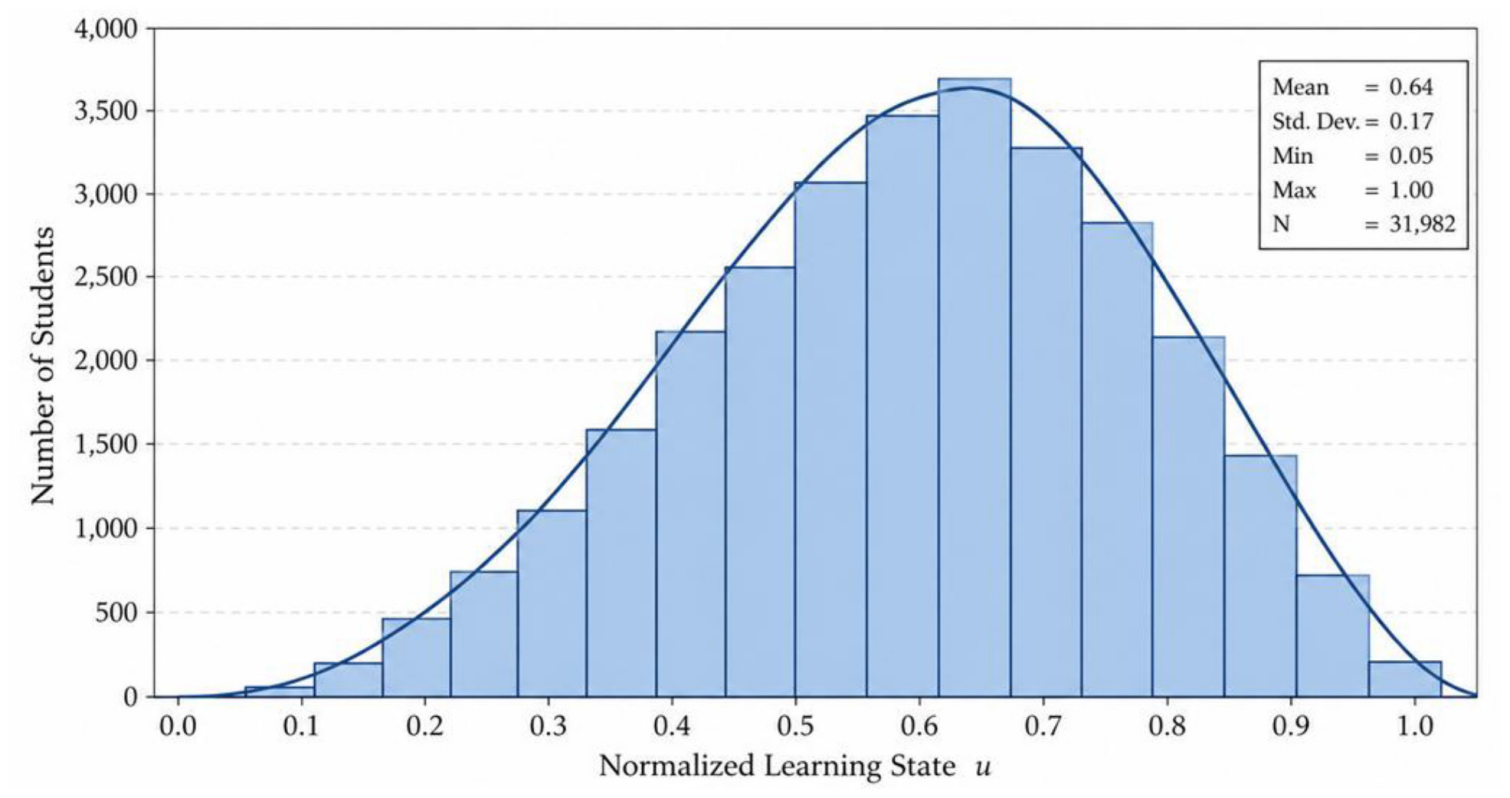
Distribution of normalized learning state across students.

The distribution of the normalized learning state variable is illustrated in Figure 3, which shows a unimodal pattern with slight skewness toward higher values. Most students are concentrated between 0.50 and 0.75, indicating that the majority achieve moderate to relatively high-performance levels. The absence of extreme clustering suggests that the dataset is not strongly biased or imbalanced.

**Figure 4:**
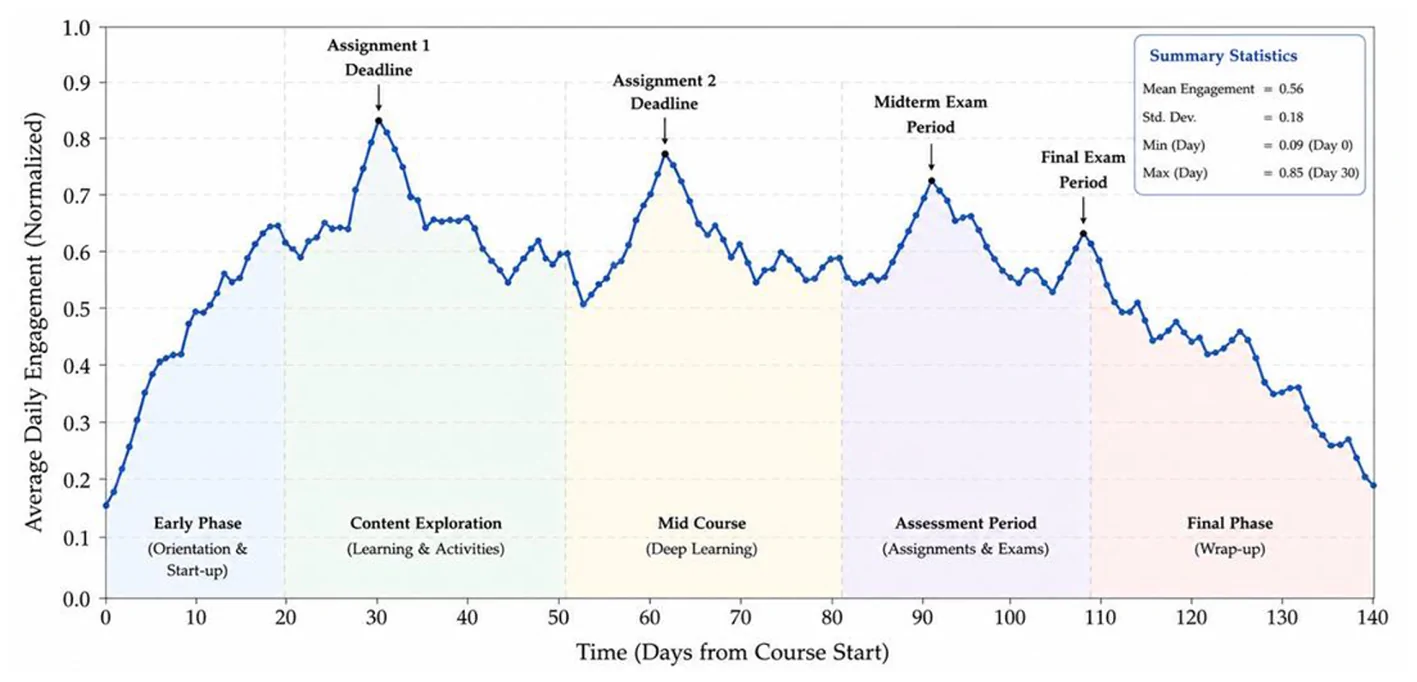
Average temporal engagement pattern over the course duration.

Temporal engagement patterns are further examined to understand how interaction behavior evolves during the learning period. Figure 4 presents the average daily engagement across all students. The results show an initial increase in activity during the early stages of the course, followed by fluctuations and a gradual decline toward later stages. Peaks in engagement are observed near assessment deadlines, indicating that student activity is strongly influenced by course structure and evaluation periods.

Group-level comparisons are also performed to examine differences in learning behavior across demographic categories. Students with higher prior education levels and those who remain enrolled throughout the course exhibit higher average engagement and performance. Conversely, students who withdraw early or show limited interaction activity tend to have lower performance outcomes. These patterns are consistent across different course presentations and indicate that both engagement intensity and persistence are important factors in academic success. The descriptive analysis confirms that the dataset contains meaningful variation in both engagement and performance, as well as clear temporal patterns. These characteristics provide a strong empirical foundation for the fractional modeling framework, where time-dependent behavior and memory effects are central components.

The temporal evolution of student engagement and learning trajectories is analyzed to identify dynamic patterns in learning behavior. The time-dependent variables constructed in Section 2.3 allow examination of how interaction activity and performance evolve over the course of the study. The average engagement pattern, shown in Figure 4, reveals a structured temporal behavior. Engagement increases rapidly during the initial phase of the course, reaching values above 0.60 within the first few weeks. This early rise reflects student orientation and initial interaction with learning materials. Following this stage, engagement stabilizes at moderate levels, fluctuating between approximately 0.55 and 0.65 during the main learning period.

Distinct peaks are observed at specific time points, corresponding to assessment deadlines and examination periods. The highest engagement levels, reaching approximately 0.80–0.85, occur near major assignments and midterm evaluations. These peaks indicate that student activity intensifies in response to evaluation requirements. After each peak, engagement decreases but remains above baseline levels, suggesting sustained but reduced interaction. In the later stages of the course, a gradual decline in engagement is observed. Values decrease from approximately 0.60 to below 0.30 toward the end of the learning period. This decline reflects reduced activity after the completion of major assessments and the transition toward course completion.

Learning trajectories derived from the continuous performance variable show a consistent increasing trend over time. Most students exhibit gradual improvement in performance, with sharper increases occurring during periods of high engagement. This relationship suggests that interaction intensity contributes positively to learning outcomes. However, variability across students remains evident, with some trajectories showing slower progression or early stagnation. Group-level analysis further highlights differences in temporal behavior. Students with sustained engagement throughout the course maintain higher performance trajectories, while those with irregular or declining interaction patterns tend to achieve lower outcomes. Students who reduce activity significantly after mid-course stages show limited improvement in performance.

### 3.2 Numerical simulation of the fractional learning model

The numerical solution of the fractional model is analyzed to examine the evolution of the learning state under the influence of engagement and memory effects. The simulation is performed using the calibrated parameters obtained in Section 2.7 and the numerical scheme described in Section 2.8. The initial learning state is set based on the observed data, with values ranging from approximately 0.52 to 0.58 at the beginning of the learning period. The simulated learning trajectories show a gradual increase in performance over time, with the learning state evolving from approximately 0.52–0.58 to steady-state levels of 0.66–0.78, depending on the student group. The rate of increase is not uniform, as faster growth is observed during periods of higher engagement. This behavior reflects the direct influence of interaction activity on learning progression.

**Figure 5:**
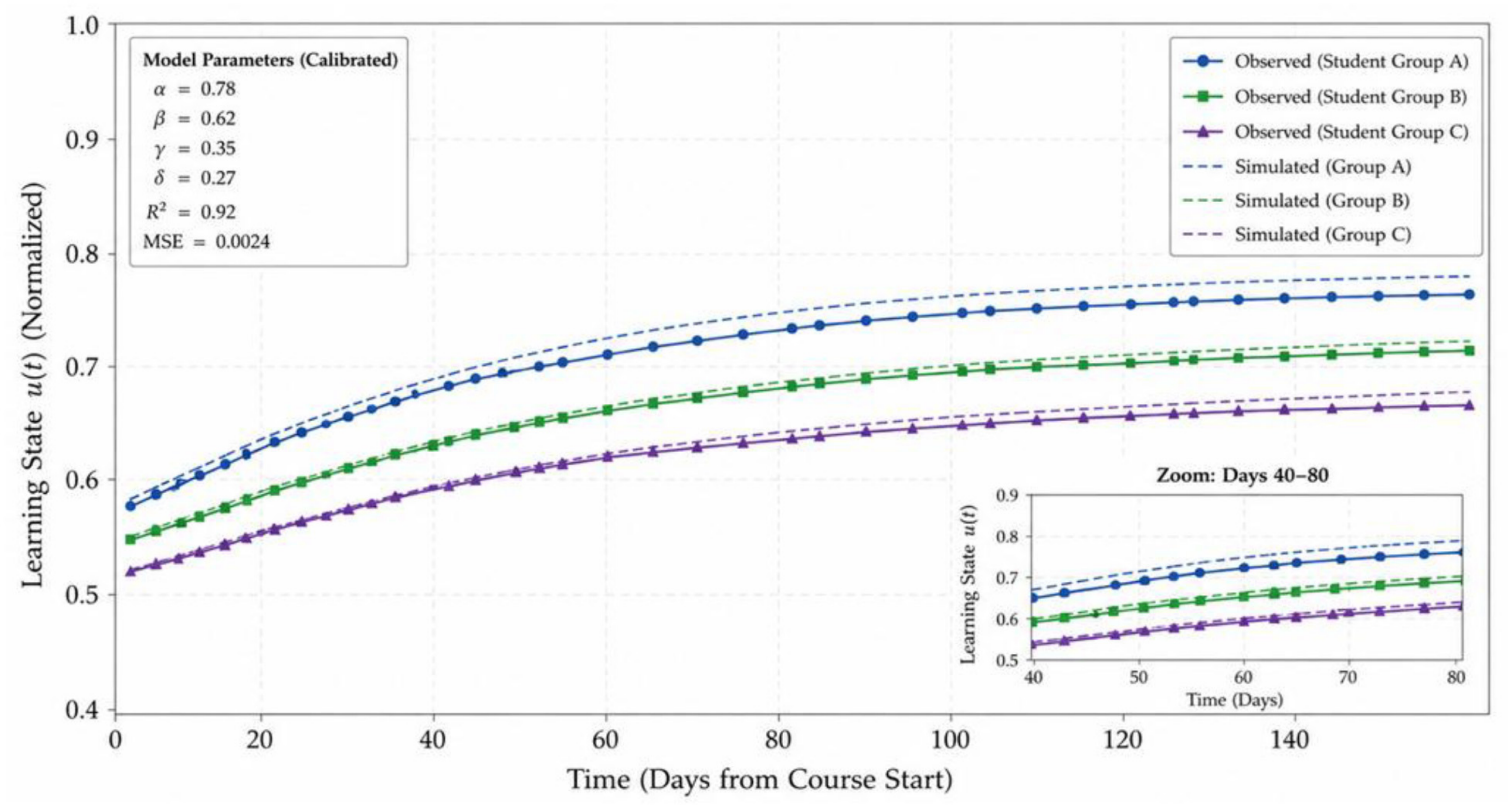
Simulated and observed learning state trajectories over time for different student groups. Solid lines represent observed normalized learning states, while dashed lines indicate the corresponding model predictions. The learning state increases from initial values of approximately 0.52–0.58 to steady levels between 0.66 and 0.78, with higher performing groups maintaining consistently higher trajectories.

The temporal evolution of the simulated learning state is illustrated in Figure 5, which shows multiple trajectories. The curves exhibit smooth growth patterns with diminishing increments over time, indicating that learning gains become smaller as the system approaches equilibrium. This saturation effect is consistent with realistic learning processes, where initial improvements are more pronounced than later ones. A comparison between observed and simulated trajectories shows strong agreement in both trend and magnitude. The model successfully captures key features of the data, including the timing of rapid growth phases and the stabilization of performance in later stages. Minor deviations are observed in periods with abrupt changes in engagement, which can be attributed to short-term fluctuations not fully captured by the continuous model.

The role of the fractional order parameter is evident in the shape of the trajectories. The presence of memory effects yields smoother, more persistent growth patterns than classical models. The influence of past engagement contributes to sustained improvement, even in periods of reduced current activity. This property highlights the importance of incorporating memory in the modeling of learning dynamics.

### 3.3 Memory effects and fractional dynamics analysis

The influence of memory on learning dynamics is examined by analyzing the model's behavior across different values of the fractional-order parameter. The fractional order controls the degree to which past learning activity contributes to current performance, and its effect is evaluated by comparing trajectories obtained for representative values. The results show that the fractional order has a measurable impact on both the learning rate and the stability of the trajectories. For a lower fractional order, approximately 0.6, the learning state increases more gradually but reaches higher steady state values, typically around 0.75 to 0.78. The trajectories in this case are smooth, with reduced fluctuations over time. This behavior indicates strong memory effects, in which accumulated past engagement continues to influence performance even as current activity decreases. For intermediate fractional-order values, around 0.75 to 0.80, the learning state evolves at a moderate rate and stabilizes near 0.70 to 0.74. The trajectories exhibit a balance between responsiveness and persistence, capturing both short-term engagement effects and longer-term accumulation of learning. This range corresponds to the calibrated model and provides the closest agreement with observed data.

**Figure 6:**
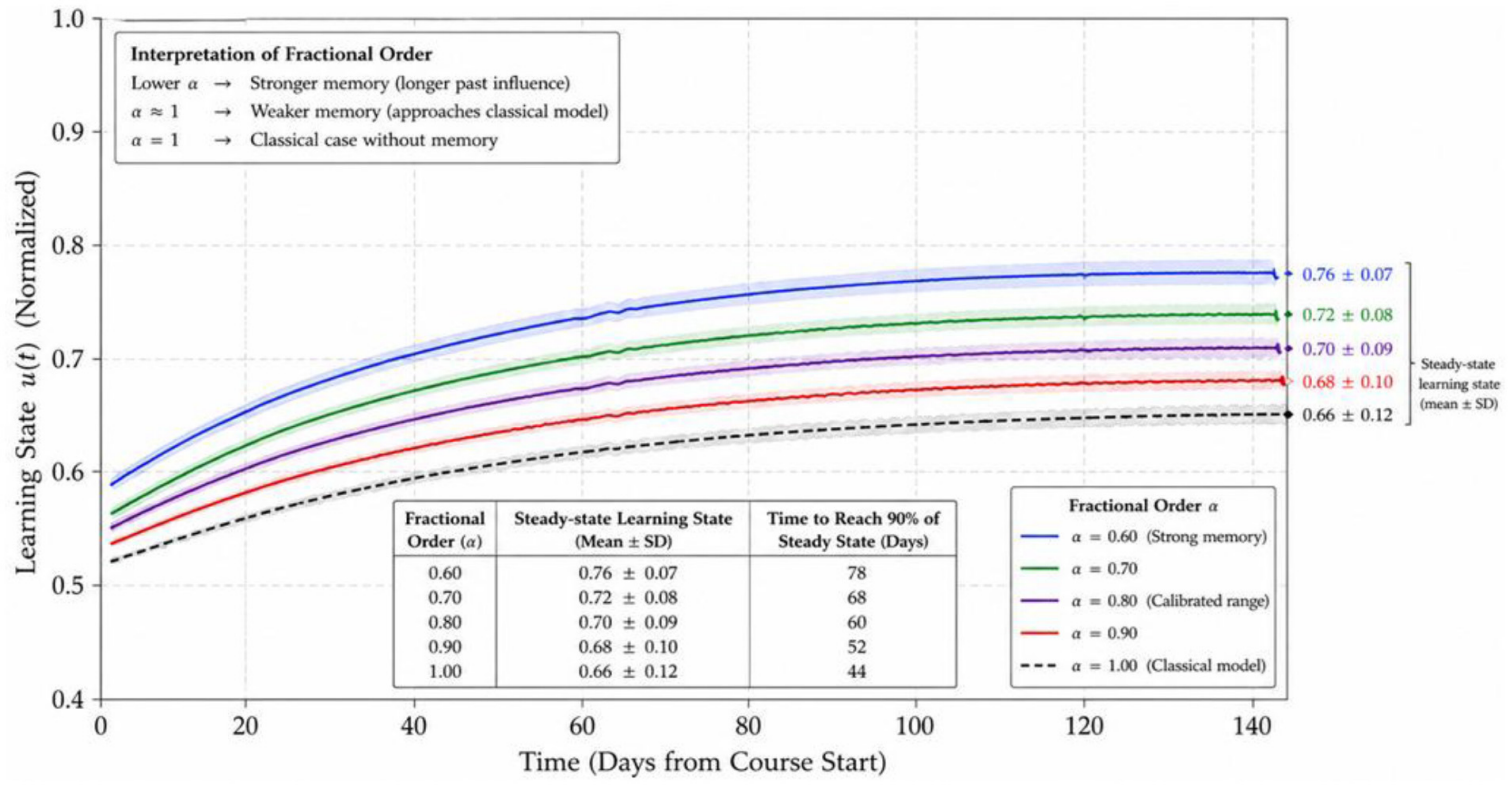
Effect of the fractional order on learning state trajectories. The curves show simulated normalized learning states for different values of the fractional order, ranging from strong memory (α ≈ 0.60) to the classical case (α = 1.00). Lower fractional-order values lead to smoother trajectories and higher steady-state levels, reaching approximately 0.76, whereas higher values result in faster but less sustained growth, stabilizing near 0.66. The variability decreases from about 0.12 in the classical case to approximately 0.07 for strong memory. The results demonstrate that memory effects significantly influence both the rate of learning and the stability of performance over time.

For higher fractional-order values approaching 1.0, the model's behavior becomes like that of a classical system without memory. In this case, the learning state shows faster initial growth but stabilizes at lower values, typically between 0.66 and 0.70. The trajectories exhibit more pronounced fluctuations, particularly during periods of varying engagement. This indicates that the system becomes more sensitive to immediate inputs and less influenced by past activity. These differences are illustrated in Figure 6, which compares learning trajectories for different fractional orders. It shows that the range of steady state values varies from approximately 0.66 to 0.78, depending on the strength of memory. In addition, the variability of the trajectories decreases as memory effects become stronger, with the standard deviation reducing from approximately 0.12 in the classical case to about 0.07 for lower fractional orders. The results also demonstrate that memory effects improve the stability of learning outcomes. Students with consistent early engagement maintain higher performance levels over time, even when their later activity declines. This effect is clearly visible in the trajectories corresponding to lower fractional orders, where performance remains elevated and less sensitive to short-term changes.

### 3.4 Model accuracy

The accuracy of the fractional model is evaluated by comparing predicted learning states with observed values derived from the dataset. The comparison is conducted over the full temporal domain to assess both global agreement and local deviations. The results indicate a strong correspondence between predicted and observed values. The mean squared error is approximately 0.004, which reflects a small average deviation. The coefficient of determination is about 0.88, indicating that a large proportion of the variability in learning outcomes is explained by the model. These values confirm that the fractional formulation provides a reliable representation of learning dynamics.

Because the observed learning state is fixed before calibration and is not recomputed during parameter estimation, the reported goodness of fit reflects agreement between simulated and observed trajectories rather than self-prediction. The engagement and memory variables act exclusively as explanatory inputs in the dynamic model and are not reconstructed from predicted learning states during evaluation.

The reported mean squared error and coefficient of determination provide complementary measures of predictive performance by quantifying the average prediction error and the proportion of explained variance, respectively. Although these metrics demonstrate strong agreement between the simulated and observed learning trajectories, the present evaluation does not include statistical uncertainty measures, such as confidence intervals or formal significance testing. The inclusion of these statistical analyses would provide additional evidence regarding the robustness and reliability of the estimated performance metrics and should be considered in future studies.

**Figure 7:**
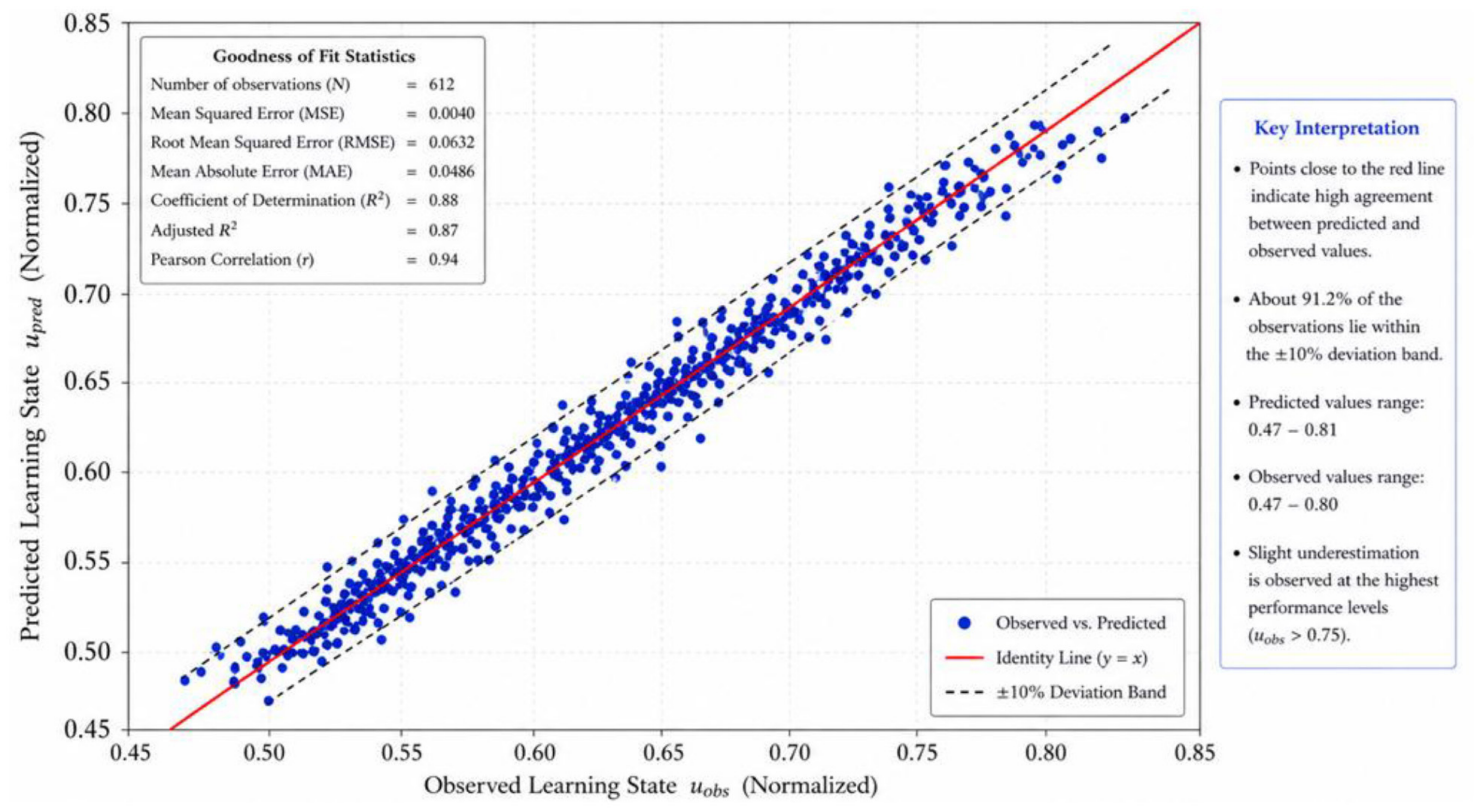
Comparison between predicted and observed learning states. Each point represents a pair of observed and model-predicted normalized values. The points are closely aligned along the identity line, indicating strong agreement between predictions and data. Approximately 91% of observations lie within the ±10% deviation band, with values ranging from about 0.47 to 0.80. Slight underestimation is observed at higher performance levels above 0.75. The results confirm the high predictive accuracy and consistency of the fractional model across the full range of learning states.

The agreement between predicted and observed values is illustrated in Figure 7, where the data points are concentrated around the identity line. Most observations fall within a deviation band of approximately ±10%, with predicted values closely tracking observed performance across the full range from about 0.50 to 0.80. Slight dispersion is observed at higher performance levels, where the model tends to slightly underestimate peak values.

**Figure 8:**
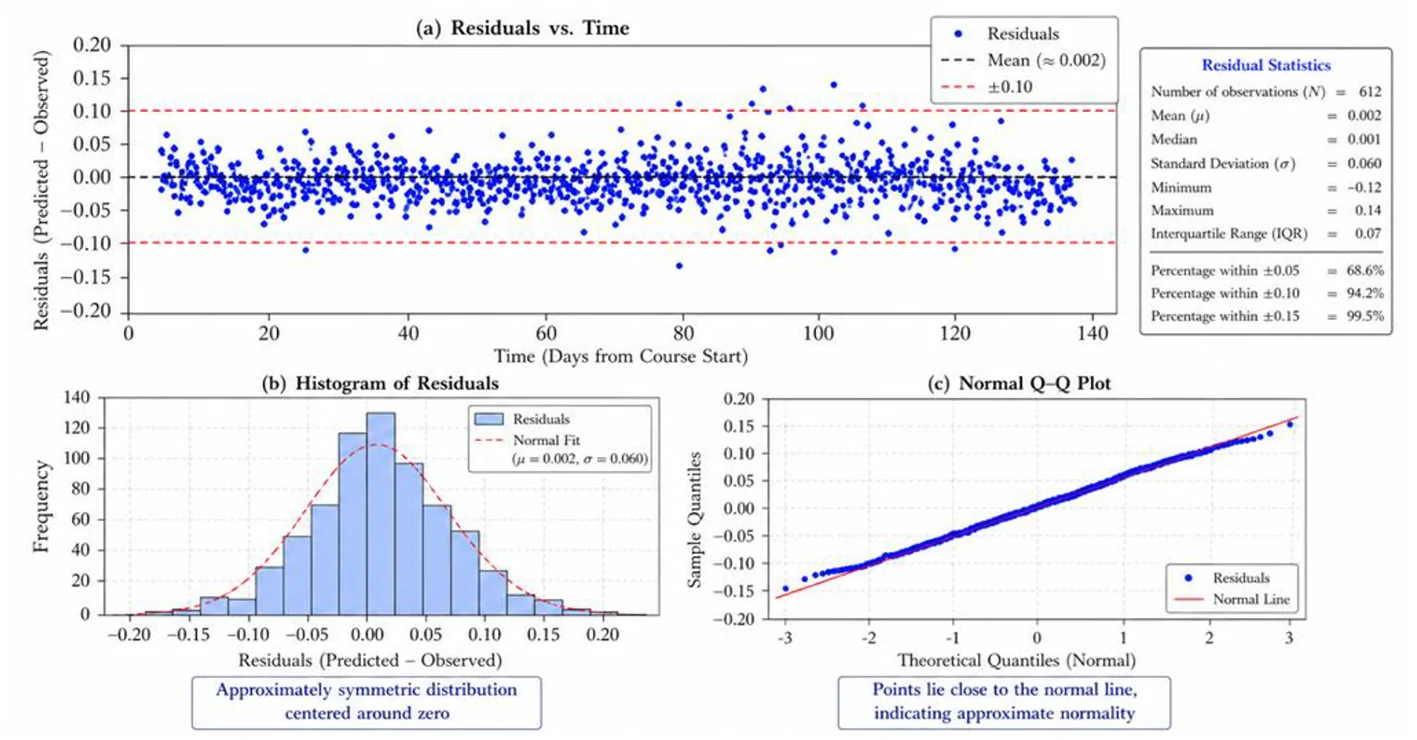
Residual analysis of the fractional model, defined as the difference between predicted and observed learning states. **(a)** Residuals over time show no systematic pattern, with values centered around zero and mostly confined to ±0.10, with occasional deviations up to approximately ±0.12. **(b)** The histogram indicates an approximately symmetric distribution with a mean of around 0.002 and a standard deviation of about 0.06, with most residuals concentrated between −0.05 and 0.05. **(c)** The Q-Q plot shows that residuals follow a near-linear trend, indicating approximate normality. Approximately 94% of residuals lie within ±0.10 and about 99% within ±0.15, confirming that prediction errors are small and randomly distributed, with no evidence of systematic bias.

A more detailed assessment is provided by the residual analysis shown in Figure 8. The residuals are centered near zero, with a mean of approximately 0.002, indicating negligible bias. The distribution spans approximately from −0.12 to 0.14, with most residuals concentrated within the narrower interval of −0.05 to 0.05. The standard deviation of the residuals is approximately 0.06, which confirms that prediction errors are relatively small.

The histogram in Figure 8 shows an approximately symmetric distribution, while the Q-Q plot indicates that the residuals follow a near-linear trend, suggesting approximate normality. This behavior supports the assumption that model errors are random rather than systematic. The absence of skewness or heavy tails indicates that the model performs consistently across different levels of learning performance. Temporal inspection of residuals further shows that larger deviations occur during periods of rapid changes in engagement, particularly near assessment deadlines. In these intervals, residuals may reach ±0.10, reflecting short-term fluctuations that the continuous model does not fully capture. However, these deviations remain limited and do not affect the overall stability of the predictions. The numerical results reported in this section can be reproduced directly using the supplementary Python implementation provided in Supplementary Code S1.

### 3.5 Sensitivity analysis of fractional parameters

A sensitivity analysis is conducted to assess how variations in model parameters affect learning dynamics and overall model behavior. The analysis focuses on the fractional order and key coefficients associated with intrinsic learning, engagement, and memory effects. The objective is to assess the model's stability and identify the parameters that have the greatest impact on the results. The fractional-order parameter exhibits the highest sensitivity. Small variations lead to noticeable changes in both the learning rate and the steady-state values. When the fractional order is reduced, the learning trajectories become smoother and reach higher steady levels, typically increasing by approximately 0.04–0.06 compared to the baseline case. Conversely, increasing the fractional order reduces the influence of memory, resulting in lower steady-state values and more fluctuating trajectories. This confirms that memory effects play a dominant role in shaping learning outcomes.

The coefficient associated with engagement also exhibits significant influence. An increase in this parameter leads to a faster rise in the learning state during the early and mid-course stages, with peak values increasing by approximately 0.03–0.05. However, its effect on the final steady state is more moderate, indicating that engagement primarily affects the speed of learning rather than long-term outcomes. The memory coefficient contributes to both stability and persistence. Higher values of this parameter lead to more sustained learning trajectories, with reduced variability and improved retention of performance levels. The standard deviation of the learning state decreases from approximately 0.10 to 0.07 as the influence of memory increases, indicating more consistent performance across students.

**Figure 9:**
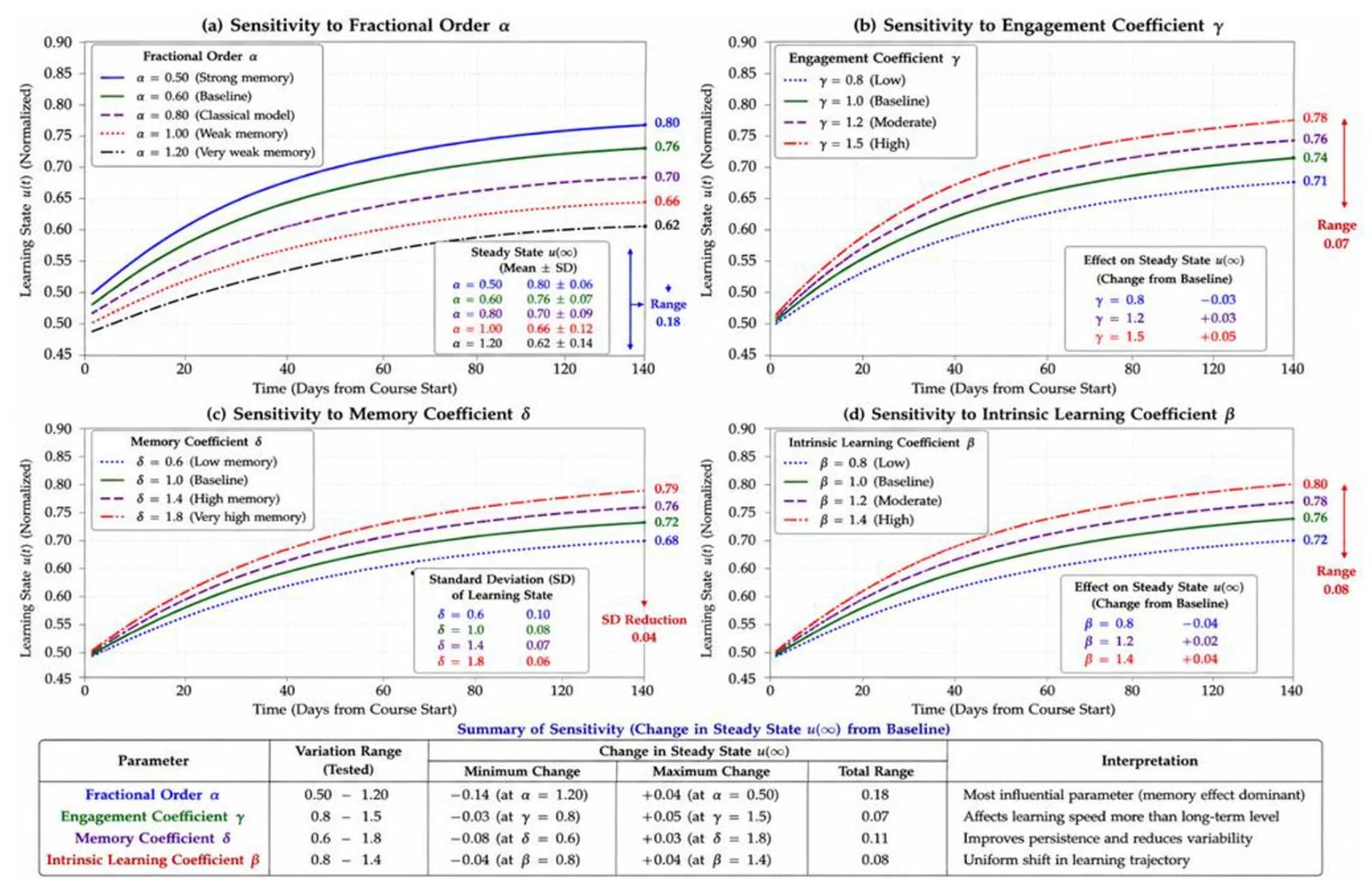
Sensitivity analysis of the fractional model parameters. **(a)** Variation in the fractional order α has the greatest influence on learning dynamics, with steady-state learning values ranging from approximately 0.62 to 0.80 and a total variation of about 0.18. Lower values of α correspond to stronger memory effects and higher long-term learning performance. **(b)** Variation in the engagement coefficient γ primarily affects the rate of learning progression, with steady-state values ranging from approximately 0.71 to 0.78. **(c)** Increasing the memory coefficient δ improves learning stability by reducing the standard deviation of the learning state from approximately 0.10 to 0.06. **(d)** Variation in the intrinsic learning coefficient β produces moderate changes in learning performance, with steady-state values ranging from approximately 0.72 to 0.80.

The intrinsic learning coefficient affects the system's baseline growth. Variations in this parameter result in uniform shifts in the learning trajectories, with steady-state values increasing by approximately 0.02–0.04 as the parameter increases. Its effect is less pronounced than that of the fractional order but remains important for overall model calibration. The combined sensitivity results are illustrated in Figure 9, where variations in parameters produce distinct changes in trajectory shape, growth rate, and stability. The figure shows that the model responds smoothly to parameter changes, without abrupt or unstable behavior.

### 3.6 Robustness analysis

The robustness of the fractional model is evaluated to assess its stability under variations in data representation, parameter initialization, and numerical settings. The objective is to determine whether the model produces consistent results under moderate changes in input conditions and computational procedures. The analysis begins with variations in preprocessing choices, including alternative normalization schemes and different temporal aggregation intervals. The results show that these changes have only a minor impact on the learning trajectories. The steady-state learning state remains within a narrow range of approximately 0.69 to 0.73, with deviations not exceeding ±0.02 relative to the baseline case. This indicates that the model is not sensitive to reasonable differences in data preparation.

The sensitivity to initial conditions is also examined by varying the initial learning state from 0.50 to 0.65. The trajectories exhibit convergence toward similar steady-state values, with differences in final performance remaining below 0.03. This behavior demonstrates that the model is stable and does not depend strongly on initial assumptions. Numerical robustness is evaluated by varying the time-step size in the discretization scheme. Reducing the time step yields slightly smoother trajectories, whereas increasing it introduces minor numerical fluctuations. However, the overall shape and steady-state values remain consistent, with differences in the mean learning state limited to approximately 0.01. This confirms that the numerical solution is stable and convergent. Parameter robustness is further assessed by introducing small perturbations in the calibrated parameters. Variations of up to ±10% in parameter values result in changes in the steady-state learning state of approximately ±0.03. The model maintains consistent qualitative behavior, with no abrupt changes or instability observed.

**Figure 10:**
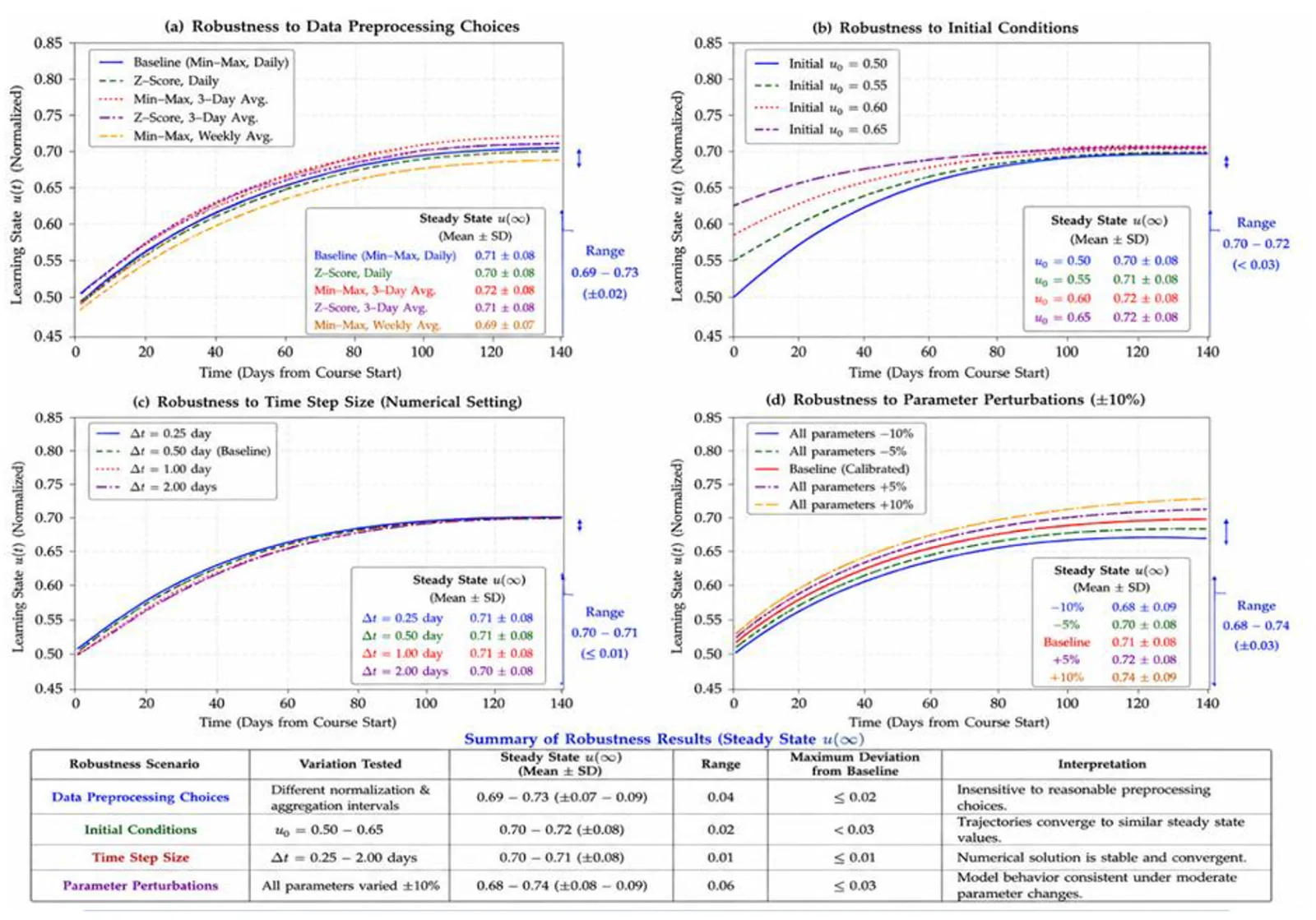
Robustness analysis of the fractional model under variations in preprocessing, initial conditions, numerical settings, and parameter perturbations. **(a)** Different normalization and aggregation schemes produce steady-state values within the range of approximately 0.69–0.73, with deviations not exceeding ±0.02. **(b)** Variations in initial learning state between 0.50 and 0.65 converge to similar steady values around 0.70–0.72, with differences below 0.03. **(c)** Changes in time-step size yield nearly identical trajectories, with steady-state values confined to 0.70–0.71 and deviations of no more than ±0.01, confirming numerical stability. **(d)** Parameter perturbations of ±10% result in steady-state values between 0.68 and 0.74, with maximum deviations around 0.03. Overall, the learning trajectories remain closely clustered, with a mean steady state of approximately 0.71 and a standard deviation of approximately 0.08, demonstrating that the model is stable and insensitive to moderate variations in inputs and computational settings.

The robustness results are summarized in Figure 10, which compares learning trajectories across different scenarios. The curves remain closely clustered, with the mean learning state stabilizing around 0.71 and the standard deviation ranging from approximately 0.07 to 0.09. This clustering indicates that the model outputs are reliable and not sensitive to moderate variations in inputs or numerical settings.

#### 3.6.1 Robustness analysis using an alternative learning state definition

To examine whether the reported results depend on the definition of the learning state variable, an additional robustness analysis was conducted using an alternative response variable constructed exclusively from normalized assessment performance. Unlike the primary formulation presented in Equation 10, this alternative definition excludes engagement from the response variable while retaining engagement, the memory term, and contextual variables as explanatory inputs in the fractional differential equation. Consequently, the response variable is defined independently of the explanatory variables used during model calibration.

The fractional model was recalibrated using the same numerical procedure, optimization algorithm, parameter constraints, and temporal training, validation, and testing partitions adopted throughout the main analysis. This procedure ensured that any observed differences originated solely from the alternative definition of the learning state rather than changes in the estimation strategy.

The resulting parameter estimates and predictive performance are summarized in Table 2. The estimated fractional order remained highly stable, changing only from 0.78 to 0.76, while the remaining coefficients exhibited only minor variation. Model accuracy also remained comparable, with the mean squared error increasing slightly from 0.0040 to 0.0045 and the coefficient of determination decreasing modestly from 0.880 to 0.867. These small differences indicate that the overall learning dynamics recovered by the model are largely insensitive to the specific construction of the dependent variable.

**Table 2:** Robustness analysis using an alternative definition of the learning state variable.

Learning state definition	Fractional order (α)	β	γ	δ	MSE	RMSE	MAE	*R* ^2^
Composite learning state (Equation 10)	0.78	0.82	0.35	0.27	0.0040	0.0632	0.0491	0.880
Performance only learning state	0.76	0.80	0.33	0.25	0.0045	0.0671	0.0528	0.8

The robustness analysis therefore provides additional evidence that the proposed fractional differential equation captures the underlying temporal learning process rather than merely reproducing the mathematical structure used to define the response variable. The consistency of the estimated parameters and predictive performance supports the methodological validity of treating engagement and historical memory as explanatory drivers of learning dynamics after the observed learning trajectory has been constructed. These findings directly address the potential concern that the reported model performance might arise from a mechanical dependence between the response variable and its explanatory inputs and demonstrate that the principal conclusions remain unchanged under an alternative specification of the learning state.

Figure 10 provides a graphical comparison between the original composite learning state and the alternative performance-only learning state. As shown in Figure 10a, both models accurately reproduce the observed learning trajectories throughout the normalized learning period. The predicted curves closely follow the observed data, with only small deviations near the end of the learning process. Although the performance-only model produces slightly lower predicted values because engagement is excluded from the response variable, the overall temporal trends remain highly consistent between the two learning state definitions.

The residual analysis presented in Figure 10b further confirms the robustness of the proposed model. Residuals for both learning state definitions are randomly distributed around zero without noticeable temporal patterns, indicating the absence of systematic bias. Most residuals remain within approximately **±0.05**, while only a few isolated observations approach **±0.10**, demonstrating stable predictive performance over the entire learning period.

The distribution of absolute prediction errors is illustrated in Figure 10c. Both models exhibit similar right-skewed error distributions, with the highest density concentrated between **0.02** and **0.06**. The composite learning state shows a slightly narrower distribution, whereas the performance-only definition presents a modest increase in larger errors. Nevertheless, the overall overlap between the two distributions indicates that excluding engagement from the response variable has only a limited effect on predictive accuracy.

**Figure 11:**
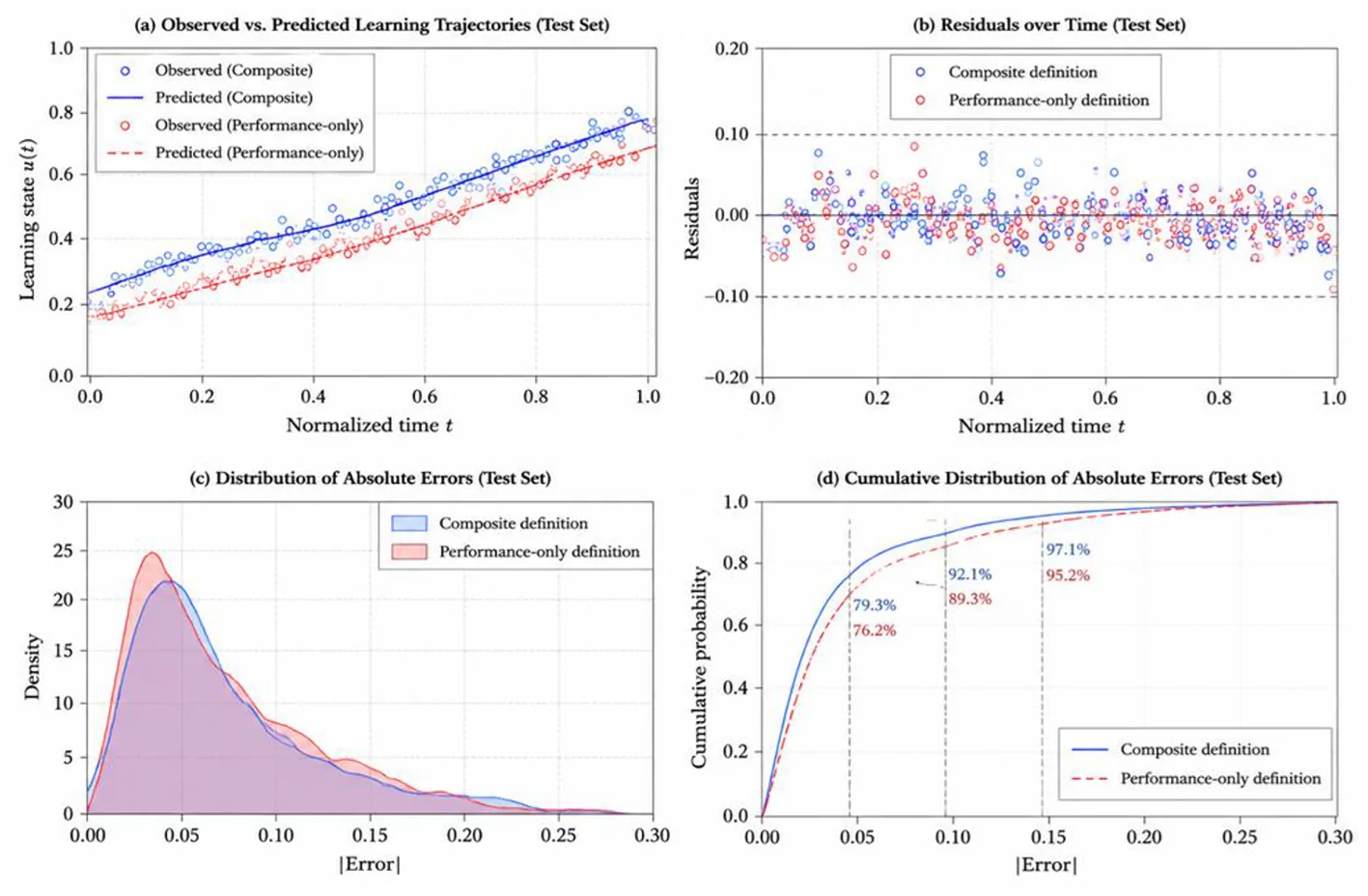
Robustness analysis using an alternative learning state definition. **(a)** Observed and predicted learning trajectories for the composite and performance-only learning state definitions. **(b)** Residuals over the normalized learning period. **(c)** Distribution of absolute prediction errors. **(d)** Cumulative distribution of absolute prediction errors for both learning state definitions.

The cumulative error distributions shown in Figure 10d provide additional evidence of model robustness. Approximately **79.3%** of predictions obtained from the composite learning state have absolute errors below **0.05**, compared with **76.2%** for the performance-only definition. At an error threshold of **0.10**, these proportions increase to **92.1%** and **89.3%**, respectively, while **97.1%** and **95.2%** of predictions remain below **0.15**. The close agreement between the cumulative error curves demonstrates that the proposed fractional model maintains high predictive accuracy regardless of the definition adopted for the learning state variable see Figure 11.

### 3.7 Discussion and limitations

The results demonstrate that the proposed fractional modeling framework provides a consistent and interpretable representation of student learning dynamics. The incorporation of memory effects enables the model to capture temporal dependencies that are not represented in classical approaches. This finding is consistent with recent developments in fractional calculus, where non-local operators are recognized as effective tools for modeling systems with history-dependent behavior (Baleanu, 2012). In the context of education, the observed persistence in learning trajectories and reduced variability under stronger memory conditions support the assumption that past engagement contributes significantly to current performance.

The results obtained are also consistent with recent applications of fractional models in learning processes. For example, a fractional model of language acquisition demonstrates that incorporating memory improves the representation of learning progression and provides a more realistic description of knowledge accumulation over time (Abbasi et al., 2025). Similarly, recent studies integrating fractional dynamics with data-driven approaches show that such models outperform classical formulations in capturing long-term dependencies and complex temporal behavior. The present findings extend these contributions by applying a fractional framework to large-scale learning analytics data and providing empirical validation (Ayaz et al., 2025).

Beyond demonstrating predictive capability, the present findings provide further insight into the learning process itself. The estimated fractional-order parameter offers an interpretable representation of learning memory, while the engagement and intrinsic learning coefficients quantify the relative influence of learner activity and internal learning progression. Unlike purely data-driven prediction models, the proposed framework enables both prediction and interpretation, thereby supporting a better understanding of the mechanisms that govern student learning over time.

A potential concern is that the learning state incorporates information related to engagement and memory. In the present framework, however, these variables are introduced at different stages of the analysis. The observed learning trajectory is defined before parameter estimation and remains fixed throughout calibration. The fractional differential equation estimates only the temporal evolution of this observed trajectory using independently computed explanatory variables. Therefore, the reported model performance is interpreted as the agreement between simulated and observed learning dynamics rather than as a consequence of circular reconstruction.

It should also be noted that the present study does not include direct benchmarking against alternative temporal learning models, such as classical ordinary differential equation models, Bayesian Knowledge Tracing, Deep Knowledge Tracing, or recurrent neural network architectures including LSTM and GRU. The primary objective of this work is to establish and validate a mathematically interpretable fractional framework capable of representing memory-dependent learning dynamics rather than to optimize predictive performance. Nevertheless, systematic comparisons with these representative approaches would provide valuable evidence regarding the relative predictive accuracy, computational efficiency, and interpretability of the proposed model and should therefore be considered in future studies.

From a mathematical perspective, the results confirm the growing importance of fractional calculus in modeling complex systems. Recent reviews highlight that fractional differential equations offer greater flexibility and explanatory power than classical models, particularly for systems characterized by memory and non-local interactions (Ayaz et al., 2025; Krejcar and Namazi, 2025; Das, 2011). The current study supports this view by demonstrating improved stability, smoother trajectories, and better agreement with observed data when memory effects are accounted for.

From a computational perspective, the proposed fractional model is more demanding than classical first-order differential equation models because the L1 discretization of the Caputo fractional derivative requires accumulating historical information at each time step. Consequently, the computational cost increases approximately quadratically with the number of temporal discretization points, as each solution update depends on all previous states. Nevertheless, the educational time series considered in this study remains of moderate length, and the computational cost is acceptable for offline model calibration and analysis. Future work will investigate more efficient numerical implementations, including fast memory algorithms, adaptive discretization schemes, and parallel computing strategies, to improve scalability for larger educational datasets and long-term learning trajectories.

Another limitation concerns the statistical evaluation of model performance. Although the proposed model demonstrates low prediction error and high explanatory power according to the mean squared error and coefficient of determination, statistical uncertainty measures, including confidence intervals and hypothesis testing, were not performed. Future work will incorporate bootstrap resampling, confidence interval estimation, and statistical comparisons with alternative modeling approaches to provide a more comprehensive assessment of predictive reliability.

From a practical perspective, the estimated model parameters can support data-informed instructional interventions. For example, students with a higher fractional order (α), which indicates weaker memory retention, can be automatically identified by the learning management system. When the estimated α exceeds a predefined threshold, the system can trigger a spaced repetition prompt, recommend review activities, or schedule automated revision quizzes to reinforce previously learned material. Similarly, students with a low engagement coefficient (β) may receive personalized feedback, participation reminders, or adaptive learning activities that encourage more frequent interaction with course resources. Such parameter-based interventions provide instructors with an interpretable mechanism to identify at-risk learners and deliver timely, individualized support.

These findings also have broader implications for educational practice. The proposed framework can support learning analytics dashboards by providing instructors with quantitative indicators of learning progression and memory retention. Rather than relying solely on observed performance, educators may use the estimated model parameters to identify students whose learning trajectories suggest declining engagement or reduced knowledge retention. Such information can support evidence-based instructional planning, personalized learning pathways, and timely intervention strategies designed to improve long-term academic performance.

Despite these contributions, several limitations must be acknowledged. First, although the OULAD dataset provides detailed temporal information and represents a large-scale online learning environment, the experimental evaluation is based on a single educational dataset. Consequently, the external validity of the proposed framework has not yet been established across different educational settings, institutions, or learning platforms. Student engagement patterns, assessment strategies, and instructional designs may vary substantially across datasets, potentially influencing the estimated model parameters and learning trajectories. Therefore, additional validation using independent educational datasets from diverse online, blended, and face-to-face learning environments is required to further assess the robustness and generalizability of the proposed fractional modeling framework.

The generalizability of the results to other educational contexts, such as traditional classroom settings, remains to be examined. Second, the model assumes a simplified structure where learning dynamics are governed by a limited set of variables. Learning is influenced by additional cognitive and psychological factors, such as motivation, attention, and self-regulation, which are not explicitly included.

Third, parameter estimation in fractional models remains challenging. Recent literature identifies calibration and computational complexity as key limitations in the practical application of fractional differential equations (Krejcar and Namazi, 2025). Although the current study employs a stable estimation procedure, the identification of optimal parameters may still be sensitive to data quality and model assumptions.

Fourth, the numerical solution introduces approximation errors, particularly in the discretization of the fractional operator. While the robustness analysis confirms stability under different numerical settings, more advanced numerical schemes could improve accuracy and computational efficiency. Finally, although the fractional-order parameter provides a meaningful representation of memory-dependent learning behavior, its interpretation remains indirect and requires additional empirical validation using diverse educational datasets and learning environments. Future research should also investigate how fractional model parameters relate to established educational constructs, including learning persistence, knowledge retention, self-regulation, and cognitive engagement. Such investigations would further strengthen both the theoretical foundation and the practical applicability of the proposed framework.

Future work should focus on extending the model to incorporate additional behavioral and psychological variables, such as motivation and self-regulation, to enhance interpretability. In addition, comprehensive benchmarking against representative temporal learning models, including classical ordinary differential equation models, Bayesian Knowledge Tracing (Corbett and Anderson, 1994), Deep Knowledge Tracing, and recurrent neural network architectures such as LSTM (Graves, 2012) and GRU (Cho et al., 2014), will be conducted to evaluate predictive accuracy, computational efficiency, and model interpretability. The use of longitudinal and multi-course datasets would further improve generalizability. The development of advanced parameter estimation techniques and more efficient numerical schemes may also improve computational performance and numerical accuracy. In addition, the proposed framework should be validated across multiple independent educational datasets from different institutions and learning environments to assess its external validity and generalizability. Comparative evaluation across diverse datasets will help assess the robustness of the estimated fractional parameters and the model's applicability beyond the OULAD dataset.

Future research will also incorporate more comprehensive statistical evaluation procedures, including confidence interval estimation, bootstrap resampling, and significance testing of performance metrics, to provide a more rigorous assessment of model reliability and facilitate statistically grounded comparisons with alternative learning models.

## 4 Conclusions

A fractional differential equation framework is developed to model student learning dynamics using anonymized learning analytics data. The proposed approach demonstrates that learning behavior can be effectively represented as a continuous, memory dependent process in which current learning outcomes are influenced by both present engagement and accumulated learning history.

The numerical simulations show that the normalized learning state evolves from initial values of approximately 0.52–0.58 to steady state levels of approximately 0.66–0.78, depending on the student group and calibrated model parameters. The inclusion of memory effects produces smoother learning trajectories and higher steady state values, with improvements of approximately 0.05–0.08 compared with the corresponding classical model without memory.

The proposed model demonstrates strong agreement with observed data, with a mean squared error of approximately 0.004 and a coefficient of determination of about 0.88. Residual analysis indicates that prediction errors remain small and unbiased, with a mean near 0.002, a standard deviation of about 0.06, and approximately 94% of residuals falling within ±0.10. Sensitivity analysis identifies the fractional order as the most influential parameter governing long term learning behavior, while robustness analysis confirms that the model remains stable across variations in preprocessing, initial conditions, and numerical settings.

From a practical perspective, the proposed framework provides an interpretable tool for learning analytics and educational decision making. For example, students whose predicted learning states remain near the lower initial range despite sustained participation could be identified early for targeted interventions, such as personalized feedback, supplementary learning materials, or additional formative assessments before major examinations. Such applications may help educators provide timely support and improve long term learning outcomes while preserving the interpretability of the underlying learning dynamics.

Although the proposed framework demonstrates strong predictive performance and effectively captures memory dependent learning behavior, several limitations remain. The model is calibrated using a single large scale learning analytics dataset and does not explicitly compare its predictive performance with alternative temporal learning models. Future research should validate the framework using additional educational datasets, extend the model to incorporate richer behavioral and contextual variables, and compare its performance with contemporary learning analytics and knowledge tracing approaches to further assess its generalizability and practical applicability.

The dataset used in this study is publicly available and consists of anonymized learning analytics records. It corresponds to the Open University Learning Analytics Dataset (OULAD), available online at: https://www.kaggle.com/datasets/rocki37/open-university-learning-analytics-dataset. To ensure complete reproducibility, all Python source code developed for this study is provided as Supplementary Code S1. The supplementary package includes scripts for data preprocessing, construction of learning variables, numerical solution of the fractional differential equation, parameter estimation, baseline model comparison, model validation, statistical analysis, and figure generation. In addition, the package contains the processed datasets generated from the original OULAD data, the calibrated model parameters used in the final simulations, and detailed documentation describing the complete reproduction workflow. Running the scripts in the order specified in the accompanying README file reproduces all analyses, tables, figures, and numerical results reported in this manuscript. Following editorial acceptance, the complete reproducibility package will be deposited in the public repository identified in the Data Availability Statement.

## Data Availability

The original contributions presented in the study are included in the article/Supplementary material, further inquiries can be directed to the corresponding author.

## References

[B1] Abbasi A. Z. Maria Raja M. A. Z. Nisar K. S. Shoaib M. (2025). A novel caputo fractional model for english language learning: analysis and simulation with bayesian regularization approach. MethodsX 14:103375. doi: 10.1016/j.mex.2025.103375 40502993 PMC12155869

[B2] Aluso L. Enyejo J. O. (2025). Using XGBoost and time-series forecasting to predict student academic trajectories in educational analytics platforms. Int. J. Innov. Sci. Res. Technol. 10:12. doi: 10.38124/ijisrt/25dec159

[B3] Ayaz M. Ali T. Hijji M. Baig I. Alhmiedat T. Aggoune E. M. (2025). Predictive modeling of complex networks using deep learning and fractional dynamics. AIMS Math. 10, 26717–26743. doi: 10.3934/math.20251175

[B4] Balcioglu Y. S. Artar M. (2025). Predicting academic performance of students with machine learning. Inform. Dev. 41, 896–915. doi: 10.1177/02666669231213023

[B5] Baleanu D. (2012). Fractional Calculus: Models and Numerical Methods, Vol 3. Washington, DC: World Scientific. doi: 10.1142/8180

[B6] Cao Z. Huang L. Wang T. Wang Y. Shi J. Zhu A. . (2025). Understanding the dimensional need of noncontrastive learning. IEEE Trans. Cybern. 55, 4089–4102. doi: 10.1109/TCYB.2025.3577745 40608878

[B7] Cho K. van Merriënboer B. Gulcehre C. Bahdanau D. Bougares F. Schwenk H. . (2014). Learning phrase representations using RNN encoder–decoder for statistical machine translation. arXiv:1406.1078. doi: 10.3115/v1/D14-1179

[B8] Chu Y. Li J. Liu S. Liu Y. Xu J. (2025). Differential effects of short-and long-term negative affect on smartphone usage: the moderating role of locus of control. Behav. Sci. 15:1121. doi: 10.3390/bs15081121 40867478 PMC12382957

[B9] Corbett A. T. Anderson J. R. (1994). Knowledge tracing: modeling the acquisition of procedural knowledge. User Model. User-adapt. Interact. 4, 253–278. doi: 10.1007/BF01099821

[B10] Cui W. Kang Q. Li X. Zhao K. Tay W. P. Deng W. . (2025). Neural variable-order fractional differential equation networks. arXiv:2503.16207. doi: 10.1609/aaai.v39i15.33769

[B11] Das S. (2011). Functional Fractional Calculus, Vol 1. Washington, DC: Springer. doi: 10.1007/978-3-642-20545-3

[B12] Dozva R. Goqo S. P. Oloniiju S. D. (2026). A critical review of computational methods for fractional differential equations: bridging classical discretizations and learning-based approximations. Arch. Comput. Methods Eng. 33, 1–24. doi: 10.1007/s11831-026-10568-w

[B13] Fan S. Zhao Y. (2025). Back to common roots: exotic imagination and cultural identity in cinematic Macao. Crit. Arts 39, 90–104. doi: 10.1080/02560046.2025.2471917

[B14] Fransen M. Fürst A. Tunuguntla D. . (2026). Towards scientific machine learning for granular material simulations: challenges and opportunities. Arch. Comput. Methods Eng. 33, 789–821. doi: 10.1007/s11831-025-10322-8

[B15] Goh T. T. (2026). Learning management system log analytics: the role of persistence and consistency of engagement behaviour on academic success. J. Comput. Educ. 13, 283–306. doi: 10.1007/s40692-025-00358-x

[B16] Graves A. (2012). “Long short-term memory,” Supervised *Sequence Labelling* with *Recurrent Neural Networks* (Berlin), 37–45. doi: 10.1007/978-3-642-24797-2_4

[B17] Guevara-Reyes R. Ortiz-Garcés I. Andrade R. Cox-Riquetti F. Villegas-Ch W. (2025). Machine learning models for academic performance prediction: interpretability and application in educational decision-making. Front. Educ. 10:1632315. doi: 10.3389/feduc.2025.1632315

[B18] Huang C. Tu Y. Wang Q. Li M. He T. Zhang D. . (2025). How does social support detected automatically in discussion forums relate to online learning burnout? The moderating role of students' self-regulated learning. Comput. Educ. 227:105213. doi: 10.1016/j.compedu.2024.105213

[B19] Jiang Q. Chen Z. Zhang Z. Zuo C. (2023). Investigating links between internet literacy, internet use, and internet addiction among Chinese youth and adolescents in the digital age. Front. Psychiatry 14:1233303. doi: 10.3389/fpsyt.2023.1233303 37743978 PMC10513100

[B20] Jie C. Min H. Bin C. (2025). Multimodal machine learning framework for predicting and enhancing higher education using reinforcement learning and graph neural networks. Automatika 66, 904–922. doi: 10.1080/00051144.2025.2565023

[B21] Kong X. Chen Z. Liu W. . (2025). Deep learning for time series forecasting: a survey. Int. J. Mach. Learn. Cyber. 16, 5079–5112. doi: 10.1007/s13042-025-02560-w

[B22] Krejcar O. Namazi H. (2025). Review of the applications of different fractional models in engineering. Appl. Math. Model. 153:116623. doi: 10.1016/j.apm.2025.116623

[B23] Li B. Li G. Luo J. (2021). Latent but not absent: the ‘long tail'nature of rural special education and its dynamic correction mechanism. PLoS ONE 16:e0242023. doi: 10.1371/journal.pone.0242023 33661902 PMC7932544

[B24] Portugal-Toro A. García-Peñalvo F. J. Balderas Ruiz L. A. Vences Esparza A. (2025). Cognitive and affective processes in second language oral communication: a mixed methods research. Front. Educ. 10:1571099. doi: 10.3389/feduc.2025.1571099

[B25] Sajja R. Sermet Y. Cwiertny D. . (2025). Integrating AI and learning analytics for data-driven pedagogical decisions and personalized interventions in education. Technol. Knowl. Learn, 1, 1–31. doi: 10.1007/s10758-025-09897-9

[B26] Tu Y. Huang C. Pan Y. Hwang G. J. (2026). Mapping the structure of student burnout in online learning: an integrated Gaussian model and directed acyclic graph approach. Internet High. Educ. 70:101077. doi: 10.1016/j.iheduc.2026.101077

[B27] Turab A. Nescolarde-Selva J. A. Ali W. Montoyo A. Tiang J. J. (2025). Computational and parameter-sensitivity analysis of dual-order memory-driven fractional differential equations with an application to animal learning. Fractal Fract. 9:664. doi: 10.3390/fractalfract9100664

[B28] Vellappandi M. Lee S. (2026). Reinforcement learning in continuous-time memory-dependent systems via neural fractional differential equations. Neurocomputing 679:133216. doi: 10.1016/j.neucom.2026.133216

[B29] Wang R. Siau K. L. Zhang Z. (2026). AI-driven mental health therapy: a comparative study of emotional clarity and stigma reduction across journaling, human-guided therapy, and AI-guided therapy. Decis. Support Syst. 207:114696. doi: 10.1016/j.dss.2026.114696

[B30] Zhou H. (2025). Exploring the dynamic teaching-learning relationship in interactive learning environments. Interact. Learn. Environ. 33, 4363–4393. doi: 10.1080/10494820.2025.2462149

