# Supplementary material for: A fractional differential equation model for student learning dynamics using anonymized learning analytics data
Source: Front Psychol. 2026 Sep 7;17:1901905. doi: 10.3389/fpsyg.2026.1901905 (PMC13590704; doi:10.3389/fpsyg.2026.1901905)
Supplement: Supplementary file 1 [file Data_Sheet_7.zip › 01_preprocessing.pdf]

```python
#!/usr/bin/env python3
# ============================================================
# Fractional Learning Dynamics
# Script 01
# Data preprocessing
# ============================================================

from pathlib import Path
import pandas as pd
from sklearn.preprocessing import MinMaxScaler

PROJECT = Path(__file__).resolve().parents[1]
RAW = PROJECT / "data" / "raw"
PROCESSED = PROJECT / "data" / "processed"
PROCESSED.mkdir(parents=True, exist_ok=True)

FILES = {
    "assessments": "assessments.csv",
    "courses": "courses.csv",
    "studentAssessment": "studentAssessment.csv",
    "studentInfo": "studentInfo.csv",
    "studentRegistration": "studentRegistration.csv",
    "studentVle": "studentVle.csv",
    "vle": "vle.csv",
}

def load():
    data = {}
    print("Loading OULAD datasets...")
    for k, v in FILES.items():
        path = RAW / v
        if not path.exists():
            raise FileNotFoundError(f"Missing file: {path}")
        data[k] = pd.read_csv(path)
        print(f"{k:20s}: {data[k].shape}")
    return data

def report_missing(data):
    print("\nMissing values")
    for name, df in data.items():
        print(f"\n{name}")
        print(df.isna().sum())

def build_master(data):
    assess = data["studentAssessment"].merge(
        data["assessments"], on="id_assessment", how="left"
    )

    assess = assess.merge(
        data["studentInfo"],
        on=["code_module", "code_presentation", "id_student"],
        how="left"
    )

    assess = assess.merge(
        data["studentRegistration"],
        on=["code_module", "code_presentation", "id_student"],
        how="left"
    )

    vle = data["studentVle"].merge(
        data["vle"], on="id_site", how="left"
    )
```

```
engagement = (
    vle.groupby(
        ["id_student", "code_module", "code_presentation", "date"],
        as_index=False
    )["sum_click"].sum()
)

# Match engagement observed up to each assessment date
assess = assess.rename(columns={"date": "assessment_date"})
engagement = engagement.rename(columns={"date": "engagement_date"})

merged = assess.merge(
    engagement,
    on=["id_student", "code_module", "code_presentation"],
    how="left"
)

merged = merged[
    merged["engagement_date"].isna() |
    (merged["engagement_date"] <= merged["assessment_date"])
]

master = (
    merged.groupby(
        ["id_student","code_module","code_presentation","id_assessment"],
        as_index=False
    )
    .agg(
        assessment_date=("assessment_date","first"),
        assessment_score=("score","first"),
        clicks=("sum_click","sum"),
        date_registration=("date_registration","first"),
        date_unregistration=("date_unregistration","first"),
        gender=("gender","first"),
        age_band=("age_band","first"),
        highest_education=("highest_education","first"),
        studied_credits=("studied_credits","first"),
        disability=("disability","first"),
        final_result=("final_result","first"),
    )
)

master["clicks"] = master["clicks"].fillna(0)
master["assessment_score"] = master["assessment_score"].fillna(
    master["assessment_score"].median()
)

scaler = MinMaxScaler()
master["performance"] = scaler.fit_transform(master[["assessment_score"]])
master["engagement"] = scaler.fit_transform(master[["clicks"]])

master["study_duration"] = (
    master["date_unregistration"] - master["date_registration"]
)
master["study_duration"] = master["study_duration"].fillna(
    master["study_duration"].median()
)

master = master.sort_values(
    ["id_student", "assessment_date"]
).drop_duplicates()

return master
```

```
def main():
    data = load()
    report_missing(data)
    master = build_master(data)

    outfile = PROCESSED / "processed_learning_data.csv"
    master.to_csv(outfile, index=False)

    print("\nProcessed dataset saved to:")
    print(outfile)

    print("\nPreview")
    print(master.head())

    print("\nSummary")
    print(master.describe(include="all"))

if __name__ == "__main__":
    main()
```
