# Supplementary material for: A fractional differential equation model for student learning dynamics using anonymized learning analytics data
Source: Front Psychol. 2026 Sep 7;17:1901905. doi: 10.3389/fpsyg.2026.1901905 (PMC13590704; doi:10.3389/fpsyg.2026.1901905)
Supplement: Supplementary file 1 [file Data_Sheet_7.zip › 03_fractional_solver.pdf]

```python
#!/usr/bin/env python3
# ============================================================
# Fractional Learning Dynamics
# Script 03
# Caputo Fractional Differential Equation Solver (L1 Scheme)
# ============================================================

from pathlib import Path
import numpy as np
import pandas as pd
from scipy.special import gamma

PROJECT = Path(__file__).resolve().parents[1]
PROCESSED = PROJECT / "data" / "processed"

INPUT = PROCESSED / "learning_variables.csv"
OUTPUT = PROCESSED / "fractional_solution.csv"

# -------- Model Parameters (initial values) --------
ALPHA = 0.80
BETA = 0.35
GAMMA = 0.45
DELTA = 0.20
DT = 1.0


def memory_term(values, alpha):
    """L1 memory coefficients."""
    n = len(values)
    coeff = np.zeros(n)
    for k in range(n):
        coeff[k] = (k + 1) ** (1 - alpha) - k ** (1 - alpha)
    return coeff


def fractional_l1_solver(u0, engagement, memory, alpha, beta, gamma_c, delta,
dt):
    """
    Solve:
    D^alpha u = beta*u + gamma*E + delta*M
    using the Caputo L1 finite difference scheme.
    """
    n = len(engagement)
    u = np.zeros(n)
    u[0] = u0

    coeff = memory_term(np.arange(n), alpha)
    factor = dt ** alpha * gamma(2 - alpha)

    for i in range(1, n):

        history = 0.0
        for k in range(1, i):
            history += coeff[k] * (u[i - k] - u[i - k - 1])

        rhs = (
            beta * u[i - 1]
            + gamma_c * engagement[i]
            + delta * memory[i]
        )

        u[i] = u0 + factor * rhs - history

    return u
```

```python
def solve_student(group):

    group = group.sort_values("assessment_date").copy()

    u = fractional_l1_solver(
        u0=group["learning_state"].iloc[0],
        engagement=group["engagement"].to_numpy(),
        memory=group["memory"].to_numpy(),
        alpha=ALPHA,
        beta=BETA,
        gamma_c=GAMMA,
        delta=DELTA,
        dt=DT,
    )

    group["predicted_learning_state"] = u
    group["residual"] = (
        group["learning_state"] - group["predicted_learning_state"]
    )

    return group


def main():

    df = pd.read_csv(INPUT)

    results = (
        df.groupby("id_student", group_keys=False)
          .apply(solve_student)
          .reset_index(drop=True)
    )

    results.to_csv(OUTPUT, index=False)

    mse = np.mean(results["residual"] ** 2)
    rmse = np.sqrt(mse)

    ss_res = np.sum(results["residual"] ** 2)
    ss_tot = np.sum(
        (results["learning_state"] -
         results["learning_state"].mean()) ** 2
    )

    r2 = 1 - ss_res / ss_tot

    print("\nFractional Solver Summary")
    print("--------------------------")
    print(f"Alpha : {ALPHA:.3f}")
    print(f"Beta  : {BETA:.3f}")
    print(f"Gamma : {GAMMA:.3f}")
    print(f"Delta : {DELTA:.3f}")
    print(f"MSE   : {mse:.6f}")
    print(f"RMSE  : {rmse:.6f}")
    print(f"R²    : {r2:.4f}")
    print(f"\nSaved: {OUTPUT}")


if __name__ == "__main__":
    main()
```
