# Supplementary material for: A fractional differential equation model for student learning dynamics using anonymized learning analytics data
Source: Front Psychol. 2026 Sep 7;17:1901905. doi: 10.3389/fpsyg.2026.1901905 (PMC13590704; doi:10.3389/fpsyg.2026.1901905)
Supplement: Supplementary file 1 [file Data_Sheet_7.zip › 06_validation.pdf]

```
#!/usr/bin/env python3
# ============================================================
# Fractional Learning Dynamics
# Script 06
# Model Validation and Performance Evaluation
# ============================================================

from pathlib import Path
import numpy as np
import pandas as pd
from sklearn.metrics import mean_squared_error, mean_absolute_error, r2_score
from scipy import stats

PROJECT = Path(__file__).resolve().parents[1]
PROCESSED = PROJECT / "data" / "processed"
RESULTS = PROJECT / "results"

RESULTS.mkdir(parents=True, exist_ok=True)

INPUT = PROCESSED / "calibrated_predictions.csv"

def temporal_split(df):
    df = df.sort_values("assessment_date")
    n = len(df)
    i1 = int(0.60 * n)
    i2 = int(0.80 * n)
    return {
        "Training": df.iloc[:i1],
        "Validation": df.iloc[i1:i2],
        "Testing": df.iloc[i2:]
    }

def evaluate(obs, pred):
    mse = mean_squared_error(obs, pred)
    rmse = np.sqrt(mse)
    mae = mean_absolute_error(obs, pred)
    r2 = r2_score(obs, pred)
    return mse, rmse, mae, r2

def main():
    df = pd.read_csv(INPUT)

    splits = temporal_split(df)

    rows = []

    for name, part in splits.items():

        obs = part["learning_state"].to_numpy()
        pred = part["prediction"].to_numpy()

        mse, rmse, mae, r2 = evaluate(obs, pred)

        residuals = obs - pred

        shapiro_p = stats.shapiro(
            residuals[:min(len(residuals),5000)]
        ).pvalue

        rows.append({
            "Dataset": name,
            "Observations": len(part),
            "MSE": mse,
            "RMSE": rmse,
```

```
            "MAE": mae,
            "R2": r2,
            "Residual Mean": residuals.mean(),
            "Residual Std": residuals.std(),
            "Residual Min": residuals.min(),
            "Residual Max": residuals.max(),
            "Shapiro p": shapiro_p
        })

    report = pd.DataFrame(rows)

    report.to_csv(
        RESULTS / "validation_metrics.csv",
        index=False
    )

    residual_table = df.copy()
    residual_table["Residual"] = (
        residual_table["learning_state"] -
        residual_table["prediction"]
    )

    residual_table.to_csv(
        RESULTS / "residuals.csv",
        index=False
    )

    print("\nValidation Summary")
    print(report)

    print("\nAverage Performance")
    print("-------------------")
    print(f"MSE  : {report['MSE'].mean():.6f}")
    print(f"RMSE : {report['RMSE'].mean():.6f}")
    print(f"MAE  : {report['MAE'].mean():.6f}")
    print(f"R²   : {report['R2'].mean():.4f}")

    print("\nFiles created")
    print(RESULTS / "validation_metrics.csv")
    print(RESULTS / "residuals.csv")

if __name__ == "__main__":
    main()
```
