# Supplementary material for: A fractional differential equation model for student learning dynamics using anonymized learning analytics data
Source: Front Psychol. 2026 Sep 7;17:1901905. doi: 10.3389/fpsyg.2026.1901905 (PMC13590704; doi:10.3389/fpsyg.2026.1901905)
Supplement: Supplementary file 1 [file Data_Sheet_7.zip › 05_baseline_models.pdf]

```
#!/usr/bin/env python3
# ============================================================
# Fractional Learning Dynamics
# Script 05
# Baseline Model Comparison
# ============================================================

from pathlib import Path
import numpy as np
import pandas as pd
from scipy.optimize import curve_fit
from sklearn.linear_model import LinearRegression
from sklearn.metrics import mean_squared_error, mean_absolute_error, r2_score

PROJECT = Path(__file__).resolve().parents[1]
PROCESSED = PROJECT / "data" / "processed"
RESULTS = PROJECT / "results"
RESULTS.mkdir(parents=True, exist_ok=True)

INPUT = PROCESSED / "learning_variables.csv"

def temporal_split(df):
    df = df.sort_values("assessment_date")
    n = len(df)
    i1 = int(0.6 * n)
    i2 = int(0.8 * n)
    return df.iloc[:i1], df.iloc[i1:i2], df.iloc[i2:]

def metrics(y, p):
    return {
        "RMSE": np.sqrt(mean_squared_error(y, p)),
        "MAE": mean_absolute_error(y, p),
        "MSE": mean_squared_error(y, p),
        "R2": r2_score(y, p)
    }

# -------- Classical ODE (alpha = 1) --------
def ode_model(t, beta, gamma, delta):
    return beta + gamma * t + delta * np.exp(-0.01 * t)

def fit_ode(train):
    t = np.arange(len(train))
    y = train["learning_state"].values
    pars, _ = curve_fit(ode_model, t, y, maxfev=10000)
    return pars

def predict_ode(df, pars):
    t = np.arange(len(df))
    return ode_model(t, *pars)

# -------- Linear Regression --------
def fit_lr(train):
    X = train[["engagement", "memory"]]
    y = train["learning_state"]
    model = LinearRegression()
    model.fit(X, y)
    return model

def evaluate(name, train, valid, test, predictor):
    rows = []
    for split_name, split in [("Train", train), ("Validation", valid), ("Test",
test)]:
        pred = predictor(split)
        m = metrics(split["learning_state"], pred)
```

```
        rows.append({
            "Model": name,
            "Dataset": split_name,
            **m
        })
    return rows

def main():
    df = pd.read_csv(INPUT)
    train, valid, test = temporal_split(df)

    ode_pars = fit_ode(train)
    ode_rows = evaluate(
        "Classical ODE",
        train,
        valid,
        test,
        lambda d: predict_ode(d, ode_pars)
    )

    lr_model = fit_lr(train)
    lr_rows = evaluate(
        "Linear Regression",
        train,
        valid,
        test,
        lambda d: lr_model.predict(d[["engagement", "memory"]])
    )

    results = pd.DataFrame(ode_rows + lr_rows)
    outfile = RESULTS / "baseline_comparison.csv"
    results.to_csv(outfile, index=False)

    print("\nBaseline Comparison")
    print(results)
    print(f"\nSaved: {outfile}")

if __name__ == "__main__":
    main()
```
