# Supplementary material for: A fractional differential equation model for student learning dynamics using anonymized learning analytics data
Source: Front Psychol. 2026 Sep 7;17:1901905. doi: 10.3389/fpsyg.2026.1901905 (PMC13590704; doi:10.3389/fpsyg.2026.1901905)
Supplement: Supplementary file 1 [file Data_Sheet_7.zip › 02_learning_variables.pdf]

```python
#!/usr/bin/env python3
# ============================================================
# Fractional Learning Dynamics
# Script 02
# Construct Time Dependent Learning Variables
# ============================================================

from pathlib import Path
import pandas as pd
import numpy as np

PROJECT = Path(__file__).resolve().parents[1]
PROCESSED = PROJECT / "data" / "processed"

INPUT = PROCESSED / "processed_learning_data.csv"
OUTPUT = PROCESSED / "learning_variables.csv"

LAMBDA = 0.05   # exponential memory decay

def compute_memory(group, lam=LAMBDA):
    """Exponential weighted memory variable."""
    memory = []
    values = group["engagement"].to_numpy()

    for i in range(len(values)):
        w = np.exp(-lam * np.arange(i, -1, -1))
        w = w / w.sum()
        memory.append(np.sum(values[:i+1] * w))
    return pd.Series(memory, index=group.index)

def cumulative_engagement(group):
    return group["engagement"].cumsum()

def engagement_intensity(group):
    days = np.arange(1, len(group) + 1)
    return group["cum_engagement"] / days

def interpolate_performance(group):
    return (
        group["performance"]
        .interpolate(method="linear")
        .fillna(method="bfill")
        .fillna(method="ffill")
    )

def build_learning_variables(df):

    df = df.sort_values(
        ["id_student", "assessment_date"]
    ).copy()

    df["performance_traj"] = (
        df.groupby("id_student", group_keys=False)
          .apply(interpolate_performance)
    )

    df["cum_engagement"] = (
        df.groupby("id_student")["engagement"]
          .transform(cumulative_engagement)
    )

    df["engagement_intensity"] = (
        df.groupby("id_student", group_keys=False)
          .apply(engagement_intensity)
```

```
    )

    df["memory"] = (
        df.groupby("id_student", group_keys=False)
          .apply(compute_memory)
    )

    # Composite learning state (matches manuscript Eq. 10)
    omega1 = 0.50
    omega2 = 0.30
    omega3 = 0.20

    df["learning_state"] = (
        omega1 * df["performance_traj"] +
        omega2 * df["engagement"] +
        omega3 * df["memory"]
    )

    return df

def summary(df):
    print("\nLearning Variable Summary\n")
    cols = [
        "performance_traj",
        "engagement",
        "cum_engagement",
        "engagement_intensity",
        "memory",
        "learning_state",
    ]
    print(df[cols].describe())

def main():
    if not INPUT.exists():
        raise FileNotFoundError(INPUT)

    df = pd.read_csv(INPUT)

    df = build_learning_variables(df)

    df.to_csv(OUTPUT, index=False)

    print(f"\nSaved: {OUTPUT}")

    summary(df)

if __name__ == "__main__":
    main()
```
