# Supplementary material for: A fractional differential equation model for student learning dynamics using anonymized learning analytics data
Source: Front Psychol. 2026 Sep 7;17:1901905. doi: 10.3389/fpsyg.2026.1901905 (PMC13590704; doi:10.3389/fpsyg.2026.1901905)
Supplement: Supplementary file 1 [file Data_Sheet_7.zip › README.pdf]

Supplementary Code S1

Python Implementation for

A Fractional Differential Equation Model for Student Learning Dynamics Using Anonymized Learning Analytics Data

---

Overview

This repository contains the complete Python implementation developed for the manuscript:

> **A Fractional Differential Equation Model for Student Learning Dynamics Using Anonymized Learning Analytics Data**

The scripts reproduce the complete computational workflow described in the manuscript, including:

- Data preprocessing
- Construction of continuous learning variables
- Fractional differential equation implementation
- Parameter estimation
- Baseline model comparison
- Model validation
- Figure generation

The implementation uses the publicly available **Open University Learning Analytics Dataset (OULAD)**.

Directory Structure

```
Supplementary_Code_S1/

│
├── README.md
├── requirements.txt
│
├── 01_preprocessing.py
├── 02_learning_variables.py
├── 03_fractional_solver.py
├── 04_parameter_estimation.py
├── 05_baseline_models.py
├── 06_validation.py
├── 07_figures.py
│
├── data/
│   ├── raw/
│   └── processed/
│
├── parameters/
│
├── results/
│
└── figures/
```

Software Requirements

Python 3.10 or newer is recommended.

Required Python packages are listed in **requirements.txt**.

Install all dependencies using

```bash
pip install -r requirements.txt
```

Input Dataset

The implementation uses the **Open University Learning Analytics Dataset (OULAD)**.

Place the original CSV files inside

```
data/raw/
```

The directory should contain

```
assessments.csv

courses.csv

studentAssessment.csv

studentInfo.csv

studentRegistration.csv

studentVle.csv

vle.csv
```

No modifications to the original files are required.

Workflow

Run the scripts in the following order.

Script 01

```
01_preprocessing.py
```

Purpose

- Load all OULAD tables
- Merge datasets
- Clean records
- Normalize variables

- Export

Output

```
processed_learning_data.csv
```

Script 02

```
02_learning_variables.py
```

Purpose

Construct

- Performance trajectory
- Engagement
- Cumulative engagement
- Engagement intensity
- Memory variable
- Learning state

Output

```
learning_variables.csv
```

Script 03

```
03_fractional_solver.py
```

Purpose

Solve the Caputo fractional differential equation using the L1 finite difference method.

Output

```
fractional_solution.csv
```

Script 04

```
04_parameter_estimation.py
```

Purpose

Estimate

- Fractional order (α)
- Learning coefficient (β)
- Engagement coefficient (γ)
- Memory coefficient (δ)

using nonlinear least squares optimization.

Outputs

```
estimated_parameters.csv

calibrated_predictions.csv
```

Script 05

```
05_baseline_models.py
```

Purpose

Compare the proposed fractional model with

- Classical ordinary differential equation
- Linear regression

Outputs

```
baseline_comparison.csv
```

Script 06

```
06_validation.py
```

Purpose

Evaluate model performance.

Metrics include

- Mean Squared Error
- Root Mean Squared Error
- Mean Absolute Error
- Coefficient of Determination
- Residual statistics

Outputs

```
validation_metrics.csv

residuals.csv
```

Script 07

```
07_figures.py
```

Purpose

Generate all figures reported in the manuscript.

Figures are saved in

```
figures/
```

Output Files

The following files are automatically generated.

```
processed_learning_data.csv

learning_variables.csv

fractional_solution.csv

estimated_parameters.csv

calibrated_predictions.csv

baseline_comparison.csv

validation_metrics.csv

residuals.csv
```

Reproducibility

Running the scripts sequentially reproduces the complete computational workflow described in the manuscript.

The implementation includes

- data preprocessing,
- numerical solution of the fractional differential equation,
- parameter calibration,
- model validation,
- baseline comparison,
- and figure generation.

Citation

If this code is used in academic work, please cite the associated manuscript.

Contact

Corresponding Author

Tao Zou

School of Management

Guangzhou College of Commerce

Email:

Tao.zou-Guangzhou@proton.me

20250351@gcc.edu.cn

License

This supplementary code is distributed solely for academic research and educational purposes.

The OULAD dataset remains subject to its original licensing conditions.
