# Supplementary material for: A fractional differential equation model for student learning dynamics using anonymized learning analytics data
Source: Front Psychol. 2026 Sep 7;17:1901905. doi: 10.3389/fpsyg.2026.1901905 (PMC13590704; doi:10.3389/fpsyg.2026.1901905)
Supplement: Supplementary file 1 [file Data_Sheet_7.zip › 07_figures.pdf]

```python
#!/usr/bin/env python3
# ============================================================
# Fractional Learning Dynamics
# Script 07
# Generate Publication Figures
# ============================================================

from pathlib import Path
import pandas as pd
import matplotlib.pyplot as plt
import numpy as np
from scipy import stats

PROJECT = Path(__file__).resolve().parents[1]
PROCESSED = PROJECT / "data" / "processed"
RESULTS = PROJECT / "results"
FIGURES = PROJECT / "figures"

FIGURES.mkdir(parents=True, exist_ok=True)

DATA = pd.read_csv(PROCESSED / "calibrated_predictions.csv")
VALID = pd.read_csv(RESULTS / "validation_metrics.csv")
BASE = pd.read_csv(RESULTS / "baseline_comparison.csv")

plt.rcParams["figure.dpi"] = 300
plt.rcParams["font.family"] = "DejaVu Sans"

# Figure 3: Distribution
plt.figure(figsize=(6,4))
plt.hist(DATA["learning_state"], bins=30)
plt.xlabel("Learning State")
plt.ylabel("Frequency")
plt.tight_layout()
plt.savefig(FIGURES / "Figure3_Distribution.png")
plt.close()

# Figure 4: Mean engagement
eng = DATA.groupby("assessment_date")["engagement"].mean()
plt.figure(figsize=(7,4))
plt.plot(eng.index, eng.values)
plt.xlabel("Assessment Day")
plt.ylabel("Mean Engagement")
plt.tight_layout()
plt.savefig(FIGURES / "Figure4_Engagement.png")
plt.close()

# Figure 5: Observed vs predicted
plt.figure(figsize=(6,6))
plt.scatter(DATA["learning_state"], DATA["prediction"], s=10)
lims=[0,1]
plt.plot(lims, lims, "r--")
plt.xlabel("Observed")
plt.ylabel("Predicted")
plt.tight_layout()
plt.savefig(FIGURES / "Figure5_Observed_vs_Predicted.png")
plt.close()

# Figure 6: Example alpha comparison
t=np.arange(100)
plt.figure(figsize=(7,4))
for a in [0.6,0.8,1.0]:
    y=1-np.exp(-(a/8)*t/10)
    plt.plot(t,y,label=f"α={a}")
plt.legend()
```

```
plt.xlabel("Time")
plt.ylabel("Learning State")
plt.tight_layout()
plt.savefig(FIGURES / "Figure6_Fractional_Order.png")
plt.close()

# Figure 7: Residuals
res=DATA["learning_state"]-DATA["prediction"]
plt.figure(figsize=(7,4))
plt.scatter(range(len(res)),res,s=5)
plt.axhline(0,color="red",linestyle="--")
plt.xlabel("Observation")
plt.ylabel("Residual")
plt.tight_layout()
plt.savefig(FIGURES / "Figure7_Residuals.png")
plt.close()

# Figure 8: QQ plot
plt.figure(figsize=(6,6))
stats.probplot(res, dist="norm", plot=plt)
plt.tight_layout()
plt.savefig(FIGURES / "Figure8_QQPlot.png")
plt.close()

# Baseline comparison
pivot=BASE.pivot(index="Dataset",columns="Model",values="R2")
pivot.plot(kind="bar",figsize=(7,4))
plt.ylabel("R²")
plt.tight_layout()
plt.savefig(FIGURES / "Baseline_R2_Comparison.png")
plt.close()

print("Figures saved to:", FIGURES)
print("\nGenerated files:")
for f in sorted(FIGURES.glob("*.png")):
    print(" -", f.name)
```
