# Supplementary material for: A fractional differential equation model for student learning dynamics using anonymized learning analytics data
Source: Front Psychol. 2026 Sep 7;17:1901905. doi: 10.3389/fpsyg.2026.1901905 (PMC13590704; doi:10.3389/fpsyg.2026.1901905)
Supplement: Supplementary file 1 [file Data_Sheet_7.zip › 04_parameter_estimation.pdf]

```python
#!/usr/bin/env python3
# ============================================================
# Fractional Learning Dynamics
# Script 04
# Parameter Estimation by Nonlinear Least Squares
# ============================================================

from pathlib import Path
import numpy as np
import pandas as pd
from scipy.optimize import least_squares
from scipy.special import gamma

PROJECT = Path(__file__).resolve().parents[1]
PROCESSED = PROJECT / "data" / "processed"
PARAMETERS = PROJECT / "parameters"

PARAMETERS.mkdir(parents=True, exist_ok=True)

INPUT = PROCESSED / "learning_variables.csv"
OUTPUT = PARAMETERS / "estimated_parameters.csv"


def l1_coeff(alpha, n):
    return np.array([(k + 1) ** (1 - alpha) - k ** (1 - alpha)
                     for k in range(n)])


def fractional_solver(obs0, engagement, memory, params, dt=1.0):
    alpha, beta, gamma_e, delta = params

    n = len(engagement)
    u = np.zeros(n)
    u[0] = obs0

    coeff = l1_coeff(alpha, n)
    factor = (dt ** alpha) * gamma(2 - alpha)

    for i in range(1, n):

        history = 0.0
        for k in range(1, i):
            history += coeff[k] * (u[i-k] - u[i-k-1])

        rhs = (
            beta * u[i-1]
            + gamma_e * engagement[i]
            + delta * memory[i]
        )

        u[i] = obs0 + factor * rhs - history

    return u


def residuals(params, df):
    res = []

    for _, g in df.groupby("id_student"):

        g = g.sort_values("assessment_date")

        pred = fractional_solver(
            g["learning_state"].iloc[0],
```

```
            g["engagement"].to_numpy(),
            g["memory"].to_numpy(),
            params
        )

        res.extend(
            g["learning_state"].to_numpy() - pred
        )

    return np.asarray(res)


def compute_metrics(obs, pred):
    mse = np.mean((obs - pred) ** 2)
    rmse = np.sqrt(mse)
    mae = np.mean(np.abs(obs - pred))
    ss_res = np.sum((obs - pred) ** 2)
    ss_tot = np.sum((obs - np.mean(obs)) ** 2)
    r2 = 1 - ss_res / ss_tot
    return mse, rmse, mae, r2


def main():

    df = pd.read_csv(INPUT)

    x0 = [0.80, 0.35, 0.45, 0.20]

    lower = [0.05, 0.0, 0.0, 0.0]
    upper = [0.99, 5.0, 5.0, 5.0]

    result = least_squares(
        residuals,
        x0=x0,
        bounds=(lower, upper),
        args=(df,),
        verbose=1
    )

    params = result.x

    predictions = []

    for _, g in df.groupby("id_student"):

        g = g.sort_values("assessment_date").copy()

        pred = fractional_solver(
            g["learning_state"].iloc[0],
            g["engagement"].to_numpy(),
            g["memory"].to_numpy(),
            params
        )

        g["prediction"] = pred
        predictions.append(g)

    pred_df = pd.concat(predictions)

    mse, rmse, mae, r2 = compute_metrics(
        pred_df["learning_state"].to_numpy(),
        pred_df["prediction"].to_numpy()
    )
```

```python
    out = pd.DataFrame({
        "Parameter": ["alpha", "beta", "gamma", "delta",
                      "MSE", "RMSE", "MAE", "R2"],
        "Value": [params[0], params[1], params[2], params[3],
                  mse, rmse, mae, r2]
    })

    out.to_csv(OUTPUT, index=False)

    pred_df.to_csv(
        PROCESSED / "calibrated_predictions.csv",
        index=False
    )

    print("\nEstimated Parameters")
    print(out)
    print(f"\nSaved parameter file: {OUTPUT}")


if __name__ == "__main__":
    main()
```
